# Physcomitrella Patens Dehydrins (PpDHNA and PpDHNC) Confer Salinity and Drought Tolerance to Transgenic Arabidopsis Plants

**DOI:** 10.3389/fpls.2017.01316

**Published:** 2017-07-26

**Authors:** Qilong Li, Xiaochen Zhang, Qiang Lv, Dong Zhu, Tianhang Qiu, Yu Xu, Fang Bao, Yikun He, Yong Hu

**Affiliations:** College of Life Sciences, Capital Normal University Beijing, China

**Keywords:** dehydrins, Physcomitrella patens, salinity stress, drought tolerance, transgenic Arabidopsis plants

## Abstract

Dehydrins (DHNs) as a member of late-embryogenesis-abundant (LEA) proteins are involved in plant abiotic stress tolerance. Two dehydrins PpDHNA and PpDHNC were previously characterized from the moss Physcomitrella patens, which has been suggested to be an ideal model plant to study stress tolerance due to its adaptability to extreme environment. In this study, functions of these two genes were analyzed by heterologous expressions in Arabidopsis. Phenotype analysis revealed that overexpressing PpDHN dehydrin lines had stronger stress resistance than wild type and empty-vector control lines. These stress tolerance mainly due to the up-regulation of stress-related genes expression and mitigation to oxidative damage. The transgenic plants showed strong scavenging ability of reactive oxygen species(ROS), which was attributed to the enhancing of the content of antioxidant enzymes like superoxide dismutase (SOD) and catalase (CAT). Further analysis showed that the contents of chlorophyll and proline tended to be the appropriate level (close to non-stress environment) and the malondialdehyde (MDA) were repressed in these transgenic plants after exposure to stress. All these results suggest the PpDHNA and PpDHNC played a crucial role in response to drought and salt stress.

## Introduction

Abiotic stressors, such as drought and salinity, can affect the normal growth of plants by affecting their physiological and metabolic processes, ultimately inhibiting the production of crops. This mainly affects the plant growth rate and tillering and finally results in the reduction of output (Munns and Tester, 2008; Rozema and Flowers, 2008; Afrasyab et al., 2010).

Stress damage is frequently reflected in the generation of reactive oxygen species (ROS), which can cause damage to cellular components if accumulation reaches a certain threshold (Miller et al., 2010; Krasensky and Jonak, 2012). ROS, such as ^1^O_2_, H_2_O_2_, ${\text{O}}_{2}^{\cdot -}$, and OH•, can cause oxidative damage to proteins, DNA, and lipids (Apel and Hirt, 2004). These superoxides can affect the stability of the structure of the latter and ultimately cause it to lose its function.

Plant biologists have long been interested in the mechanisms underlying the responses of plants to environmental changes, and a number of regulatory and/or protective proteins have been identified in plants exposed to different stressors (Choi et al., 1999; Skinner et al., 2005; Svensson et al., 2006; Yamaguchi-Shinozaki and Shinozaki, 2006). Dehydrin proteins (DHNs) are characteristic of such proteins, and confer outstanding ability to resist stress during periods of drought and salinity stress as well as in cold environments (Close et al., 1989; Porat et al., 2004; Rorat et al., 2006; Tripepi et al., 2011).

DHNs are highly hydrophilic proteins that belong to the group II (also called D-11) Late Embryogenesis Abundant (LEA) family that accumulate in the late stages of embryogenesis, during the period in which the moisture content of the seed is decreased, and also in response to various types of stress (Liii, 1993; Close, 1996, 1997; Battaglia, 2008). These proteins, which are characterized by conserved K, Y, and S segments (Close, 1996), rendering them highly conserved in evolution, are found in a variety of plants. As reported previously, the specific repetitive sequences and K-rich segments are obvious characteristics of the DHN group of the *Arabidopsis* LEA protein family (Dure et al., 1989; Close, 1996). Only the K segment (EKKGIME/DKIKEKLPG) is found in almost all the DHNs. These conserved motifs, which are thought to form an amphipathic α-helix structure (Liii, 1993), protect plant cells by interacting with both membranes and partially denatured proteins (Close, 1996; Mouillon et al., 2006; Rahman et al., 2010).

The Y segment (DEYGNP) is usually found in 1–3 copies in the N-terminal region of these proteins. The S segment in DHNs is mainly composed of 5–7 serine residues followed by three acidic amino acids, usually located near the C-terminus. The main function of these segments is related to nuclear localization (Jensen et al., 1998). DHNs are classified into five subclasses according to the type and number of these conserved/identifiable motifs: YnSK2, Kn, SKn, Y2Kn, and KnS (Mundy and Chua, 1988).

DHN genes are highly expressed under conditions of various types of stress, such as drought, cold, or high salinity (Yoon et al., 2009; Agarwal et al., 2016). Under such stressful conditions, DHNs accumulate in most tissues and cells and are mainly distributed in the nucleus and cytoplasm of the plant cell. Previous studies indicated that DHNs interact with the chloroplast (Mueller and Fernando, 2003; Tunnacliffe and Wise, 2007), mitochondria (Borovskii et al., 2002; Grelet, 2004), tonoplast (Heyen et al., 2002), endoplasmic reticulum (ER) (Ukaji et al., 2001), cytosol (Roberts et al., 1993), and nuclei (Liu et al., 2013). Some DHNs were found to accumulate in the root tip, open stomata, and cells surrounding the vascular tissue, suggesting that these proteins may play roles in these special tissues under non-stress conditions (Nylander et al., 2001).

The accumulation of DHNs is frequently associated with responses to stress. Studies regarding the functions of DHN3 and DHN9 in barley showed that the expression levels of these proteins were positively correlated with the chlorophyll a and b contents and had a significant effect on osmotic adjustment. They were also correlated with low levels of malondialdehyde (MDA), an indicator of plant oxidative stress, as well as reduced electrolyte leakage (Karami et al., 2013). A DHN gene knockout *P. patens* mutant showed poorer stress resistance and recovery from salt and osmotic pressure compared to the wild-type (WT), indicating that DHNs are indispensable for the alleviation of cell dehydration (Saavedra et al., 2006; Ruibal et al., 2012). Furthermore, there is increasing evidence that DHN transgenic plants show enhanced stress resistance. For example, *Nicotiana tabacum* transgenic plants overexpressing the citrus unshiu COR19 gene showed effective resistance to cold stress (Hara et al., 2003). Additionally, transgenic *Oryza sativa* overexpressing both *Hordeum vulgare* HVA1 (Xu et al., 1996) and wheat PMA80 and PMA1959 genes (Cheng et al., 2002) showed increased tolerance to both water deficit and salinity. Overexpression of DHN-5 of *Triticum durum* in *Arabidopsis* transgenic plants resulted in enhanced tolerance to osmotic stress (Brini et al., 2010). Thus, research regarding DHN transgenic plants with regard to the cultivation of stress-resistant mutants and further studies of the DHN family have far-reaching implications in plant biology.

DHNs have been found in all higher plants, including angiosperms and gymnosperms (Close, 1997), and there have also been reports regarding the existence of proteins similar to DHNs in lower land plants (Velten and Oliver, 2001). However, there have been few reports regarding heterologous expression of DHNs from lower plants in higher plants. Here, we selected the DHN genes of the moss *Physcomitrella patens*, which is highly tolerant to dehydration, salinity, low temperature, and osmotic stress, making it an ideal material for research on stress tolerance (Frank et al., 2005; Charron and Quatrano, 2009). *Physcomitrella patens* encodes fewer DHN-like proteins than seed plants (Choi et al., 1999; Svensson et al., 2002). LEA protein is highly represented in *Arabidopsis thaliana*, and 51 LEA genes as well as 10 DHN genes have been identified in this species (Biesethève et al., 2008; Hundertmark and Hincha, 2008). Therefore, we generated *A. thaliana* mutants by heterologous expression of *P. patens* DHN genes and analyzed their stress tolerance. The results indicated that the transgenic plants could effectively resist drought and salt stress due to their enhanced antioxidant capacity.

## Results

### PpDHNA and PpDHNC are parts of DHN proteins

**Figure 1:**
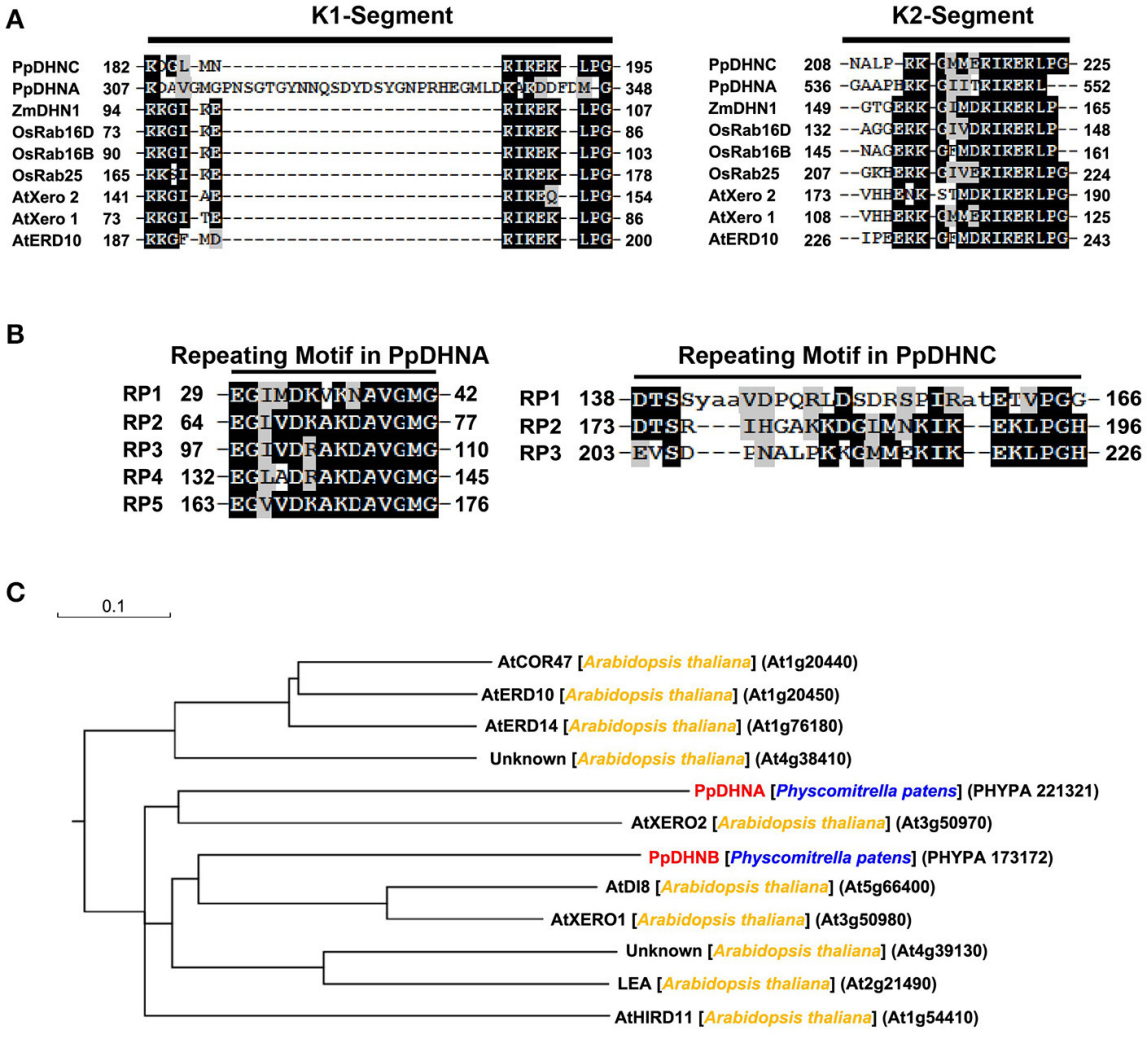
Analysis of PpDHNA (Phypa 221321) and PpDHNC (Phypa 173172) proteins. **(A,B)** Alignment of the K segment and repeating motifs found in the PpDHNA and PpDHNC protein sequence using RADAR (http://www.ebi.ac.uk/Tools/pfa/radar/). Motifs are labeled RP1 to RP5 and numbers refer to the amino acid position of the first residue. **(C)** Phylogenetic relationships between PpDHNA and PpDHNC protein and related proteins from *Arabidopsis thaliana*.

The DHN proteins, members of the LEA protein family, are characterized by their extreme hydrophilicity (Wise and Tunnacliffe, 2004; Figure S1). Their biased amino acid composition also results in stability in response to heat in solution, which is similar to the recently developed concept of “hydrophilins” (Garayarroyo et al., 2000). The K segment (Figure 1A) as the key position of LEA proteins to bind the membrane (Koag et al., 2003, 2009), which plays a significant role in the cold tolerance effect of DHNs (Eriksson et al., 2011). The results of amino acid sequence analysis indicated the presence of five tandemly repeated motifs in PpDHNA and of three in PpDHNC (Figure 1B) Of course, these repeated amino acid segments are also found in other DHNs (Houde et al., 1992; Neven et al., 1993; Welin et al., 1994). Furthermore, we compared PpDHN with 10 different DHN proteins from *Arabidopsis* (Hundertmark and Hincha, 2008) and divided them into different groups according to their sequence (Figure 1C). The results indicated that the PpDHN proteins are closely related to the DHNs of *Arabidopsis* (Figure S2 and Table S1).

### Subcellular localization of PpDHNA and PpDHNC

**Figure 2:**
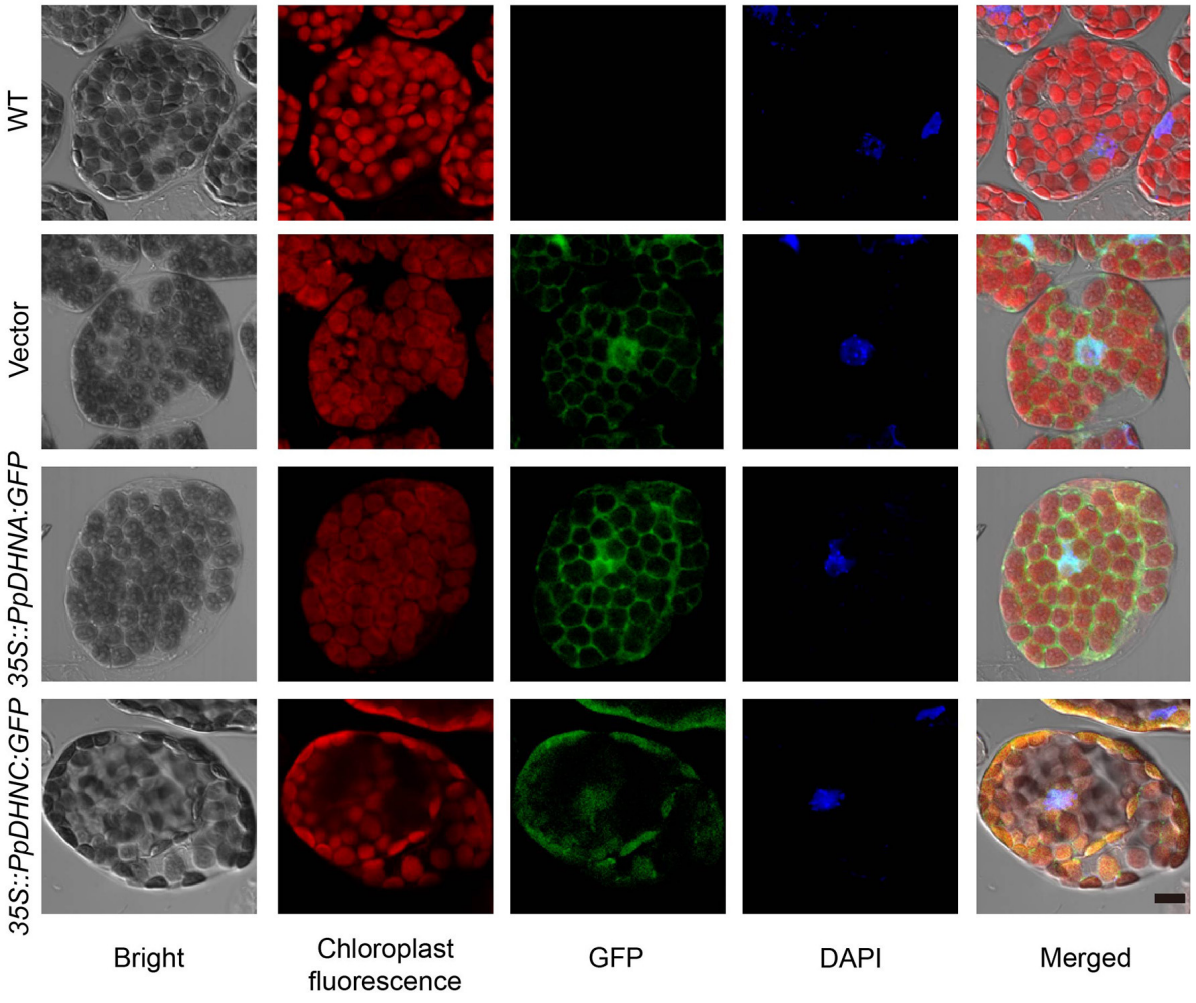
Subcellular localization of 35S::PpDHN::GFP fusion proteins in transgenic Arabidopsis. Subcellular distribution of the 35S::PpDHN::GFP fusion proteins in epidermis leaf cells using confocal laser scanning microscope. DAPI (4′,6-diamidino-2- phenylindole) binds strongly to A-T rich regions of DNA in nuclei. The red is chloroplast fluorescence, the green is GFP and the blue is DAPI fluorescence. Scale bar = 1 μm.

For the majority of LEA proteins, both the LEA-GFP and the corresponding GFP-LEA protein fusions were routinely found to give a specific fluorescent pattern typical of a cytosolic location. As indicators of the subcellular localizations of PpDHNA and PpDHNC, the fusion proteins 35S::PpDHNA::GFP mostly accumulated in the cytosol and nuclei, and the 35S::PpDHNC::GFP fusion protein showed more prominent localization in the chloroplast (Figure 2). Thus, it was suggested that PpDHNC conferred tolerance to the chloroplast (Artus et al., 1996). These observations suggest how the DHN proteins confer stress resistance.

### Drought and osmotic stress assay in PpDHNA and PpdHNC plants at the seedling stage

There is accumulating evidence that the heterologous expression of DHNs confers significant stress tolerance (Saibi et al., 2015b). To examine the stress tolerance effects of PpDHNA and PpDHNC, we generated transgenic plants by T-DNA insertion of 35S::PpDHNA::GFP, pLEA::PpDHNA::GFP, pLEA::PpDHNA, 35S::PpDHNC::GFP, pLEA::PpDHNC::GFP, and pLEA::PpDHNC into *Arabidopsis* (Figure S4A).

**Figure 3:**
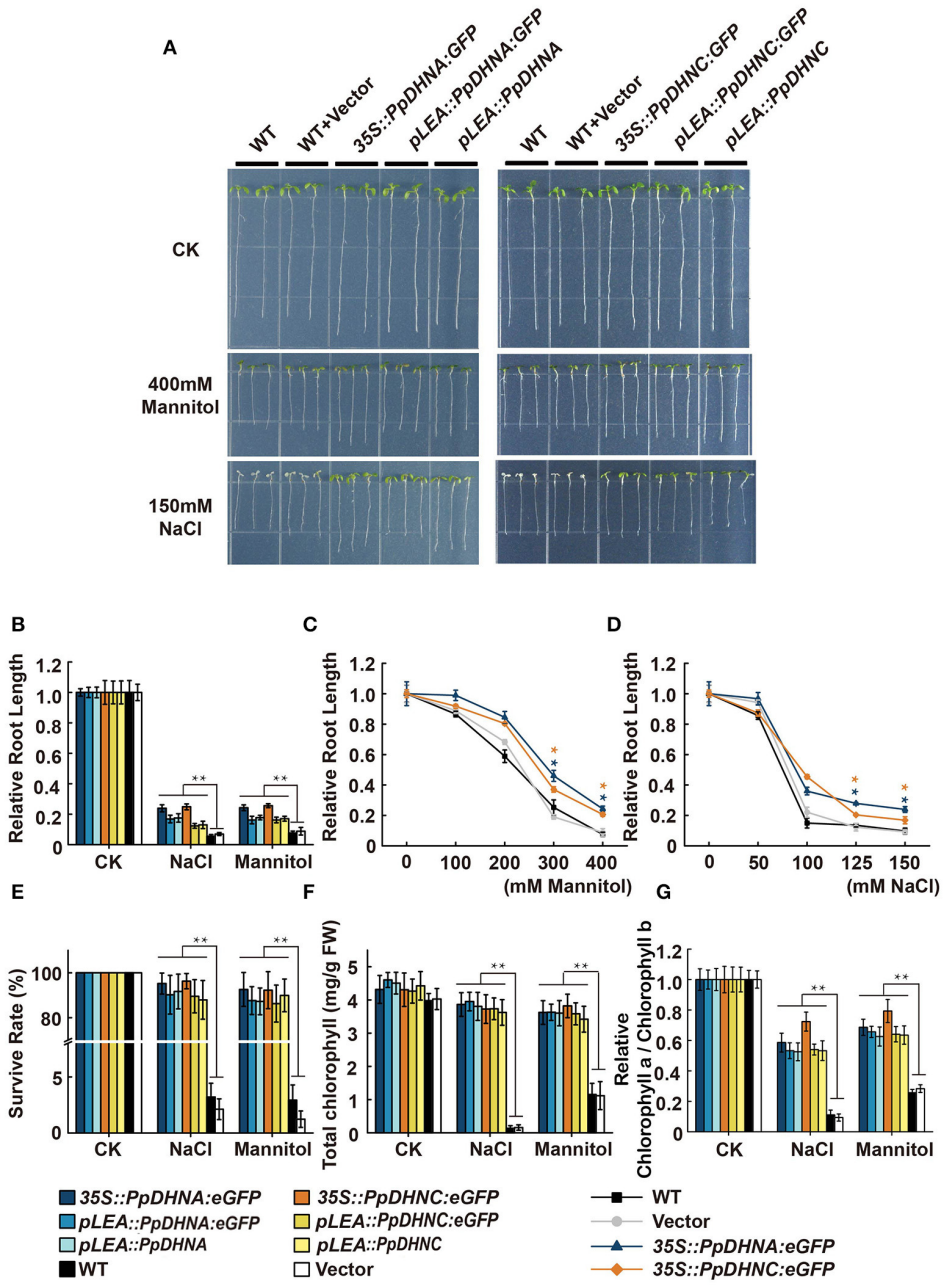
Effects of salinity and osmotic stresses on the PpDHNA and PpDHNC transgenic Arabidopsis seedlings. **(A)** Phenotypic comparison of wild type and PpDHN transgenic seedlings treated as indicated for 7 days. **(B)** Relative root length of plants treated as in **(A)**. Data are measurement from 10 seedlings ±SD, with three biological replicates for each sample. **(C,D)** Relative root length of plants treated with different concentrations of mannitol or NaCl. **(E)** Survival rate of WT and transgenic after salinity and osmotic stresses as in **(A)**. Survival rate (%) was mean of three biological replicates (*n* = 30) with ±SD value. **(F,G)** Chlorophyll contents and Chlorophyll a/Chlorophyll b of WT and transgenic after salinity and osmotic stresses as in **(A)**. The experiment was carried out in triplicate with more than 30 plant for each background. Data are mean values ±SD. Data are statistically analyzed with one-way ANOVA (LSD and Tamhane). Asterisks indicate significant different (^*^P < 0.05; ^**^P < 0.01).

The ability to adapt to osmotic or salt stress treatments was monitored in PpDHN transgenic and WT plants (seedlings growth on 1/2 MS media for 1 week) exposed for 7 days to concentrations ranging from 100 to 400 mM mannitol or 50 to 150 mM NaCl, respectively (Figure 3 and Figure S4). The PpDHNA and PpDHNC seedlings did show tolerance to the above stress treatments. Instead, the inhibition of primary root growth and substantial bleaching of cotyledons were seen in both WT and vector (Figure 3A and Figure S4). The critical concentrations for inhibition of plant growth were 100 mM for mannitol and 50 mM for NaCl, and the effects were concentration-dependent. After treatment, we confirmed PpDHNA and PpDHNC expression in transgenic plants and WT controls. The overexpressing lines showed significant improvement in the expression of RNA level. Moreover, the transgenic plants using the LEA promoter showed mild improvement in stress tolerance after treatment (Figures S3, S4B,C).

Until a concentration of 400 mM mannitol or 150 mM NaCl was reached, there were significant differences between the transgenic plants and WT controls; most transgenic plants survived, whereas the WT did not (Figure 3E). We further confirmed the phenotypes under conditions of salinity and osmotic stresses by determining chlorophyll contents. We expected that all the transgenic plants would maintain a high chlorophyll content and chlorophyll a/b ratio under the stress conditions (Figures 3F,G). Both strains showed quite different levels of stress response, manifested in the persistent growth phenotypes of the transgenic plants under conditions of stress.

### PpDHNA and PpDHNC confer salinity and drought stress tolerance to *Arabidopsis*

**Figure 4:**
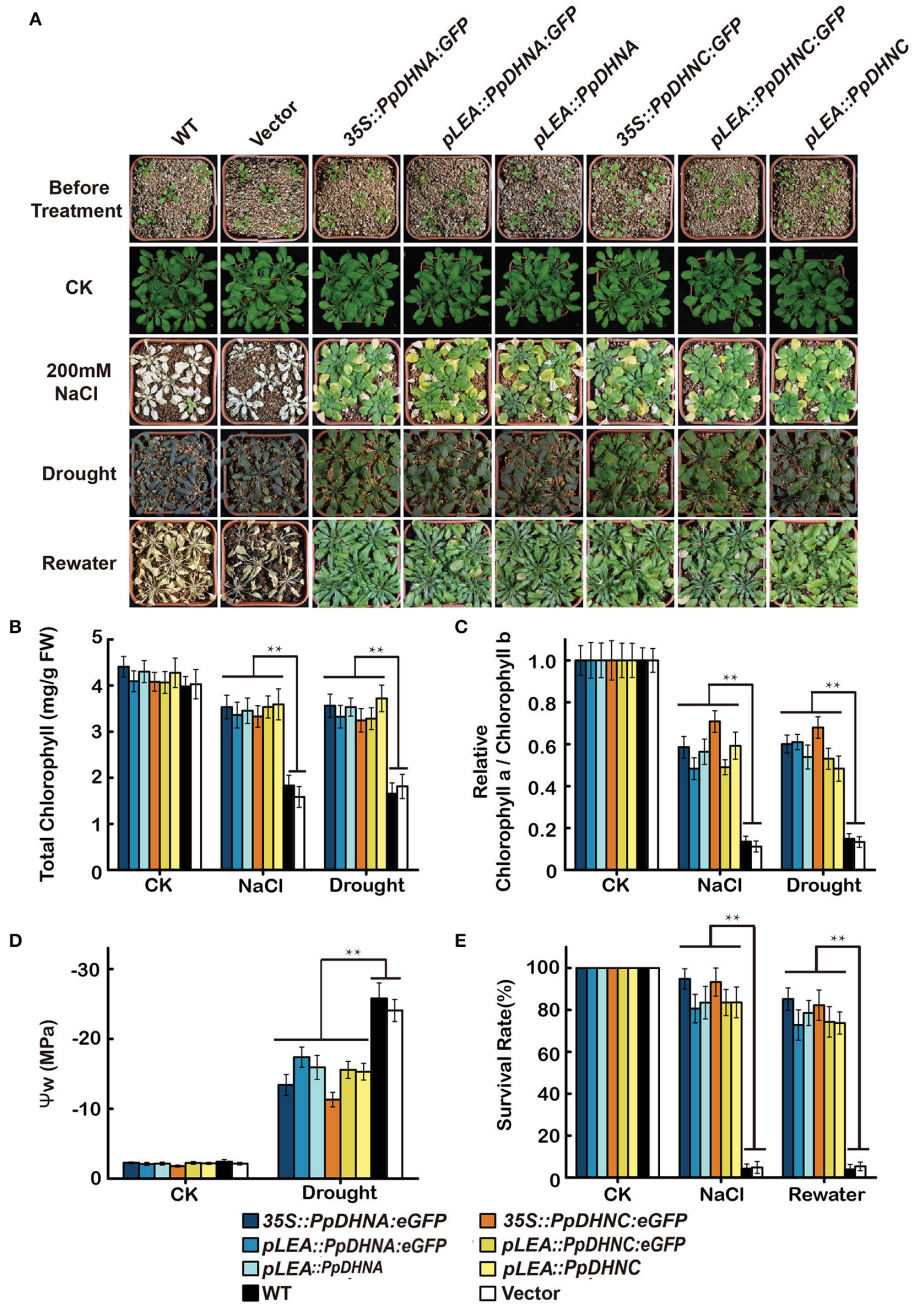
Effect of salinity and drought stresses on the PpDHNA and PpDHNC transgenic Arabidopsis plants. **(A)** Phenotypic comparison of 3-week-old wild type and PpDHN transgenic plants treated as indicated for 4 weeks. **(B,C)** Chlorophyll contents and Chlorophyll a/Chlorophyll b of 4-week plants treated as in **(A)** with 200 mM NaCl or without watering for 2 weeks. Data are means of measurement from three rosette leaves ±SD, with three replicates for each sample. **(D)** Measurement of plant water potential of plants under drought stress as in **(A)**. **(E)** Survival rate of WT and transgenic after salinity and drought stresses as in **(A)**. The experiment was carried out in triplicate with more than 30 plant for each background. Data are statistically analyzed with one-way ANOVA (LSD and Tamhane). Asterisks indicate significant different (^*^P < 0.05; ^**^P < 0.01).

The overexpression of genes encoding DHN proteins confers stress tolerance to transgenic plants, including tolerance to drought (Xu et al., 1996; Sivamani et al., 2000), dehydration (Cheng et al., 2002), and cold (Artus et al., 1996; Hara et al., 2003; Houde et al., 2004). Similarly, we characterized the phenotypes of PpDHNA and PpDHNC lines via whole-plant stress assay in pots (Figure 4A). The seedlings of these lines were transferred to well-watered soil in pots, and watering was withheld for approximately 2 weeks to gradually reduce water content. We chose 200-mM NaCl treatment as salinity stress. Under normal conditions, there were no differences in the growth of the transgenic lines and the WT controls. After induction of salinity stress, almost all the WT plants showed severe reduction of growth (Figure 4E). We observed severe dehydration of the leaves, and the whole plant was wilted (Figure 4E). In contrast, although some of the leaves of most DHN transgenic plants showed chlorosis, as a whole, they showed resistance to salinity stress and were still able to grow. Salinity tolerance in plants is often related to the accumulation of various molecules, such as sugars, including sucrose and trehalose; amino acids, especially proline in plant leaves; and also changes in chlorophyll level (Ben Rejeb et al., 2014). The chlorophyll content and the chlorophyll a/b ratio indicated that the DHN transgenic plants can carry out photosynthesis under conditions of stress (Figures 4B,C).

Meanwhile, DHN transgenic plants also showed characteristics of drought tolerance. After drought treatment, the majority of WT plants exhibited obvious wilting due to severe water deficit. In contrast, most of the PpDHN-overexpressing plants did not show any obvious wilting, and the leaves remained green. The water potential (ψ) of the PpDHN-overexpressing plants tended to be more consistent than that of the WT controls (Figure 4D) water potential. After re-watering for 3 days, the PpDHN lines were largely restored, and the degree of restoration was considerably higher than that of the WT controls (Figure 4E). These results indicated that PpDHN enhances plant tolerance to salinity and drought stress.

### Overexpression of PpDHNA and PpDHNC induces expression of stress-responsive genes

**Figure 5:**
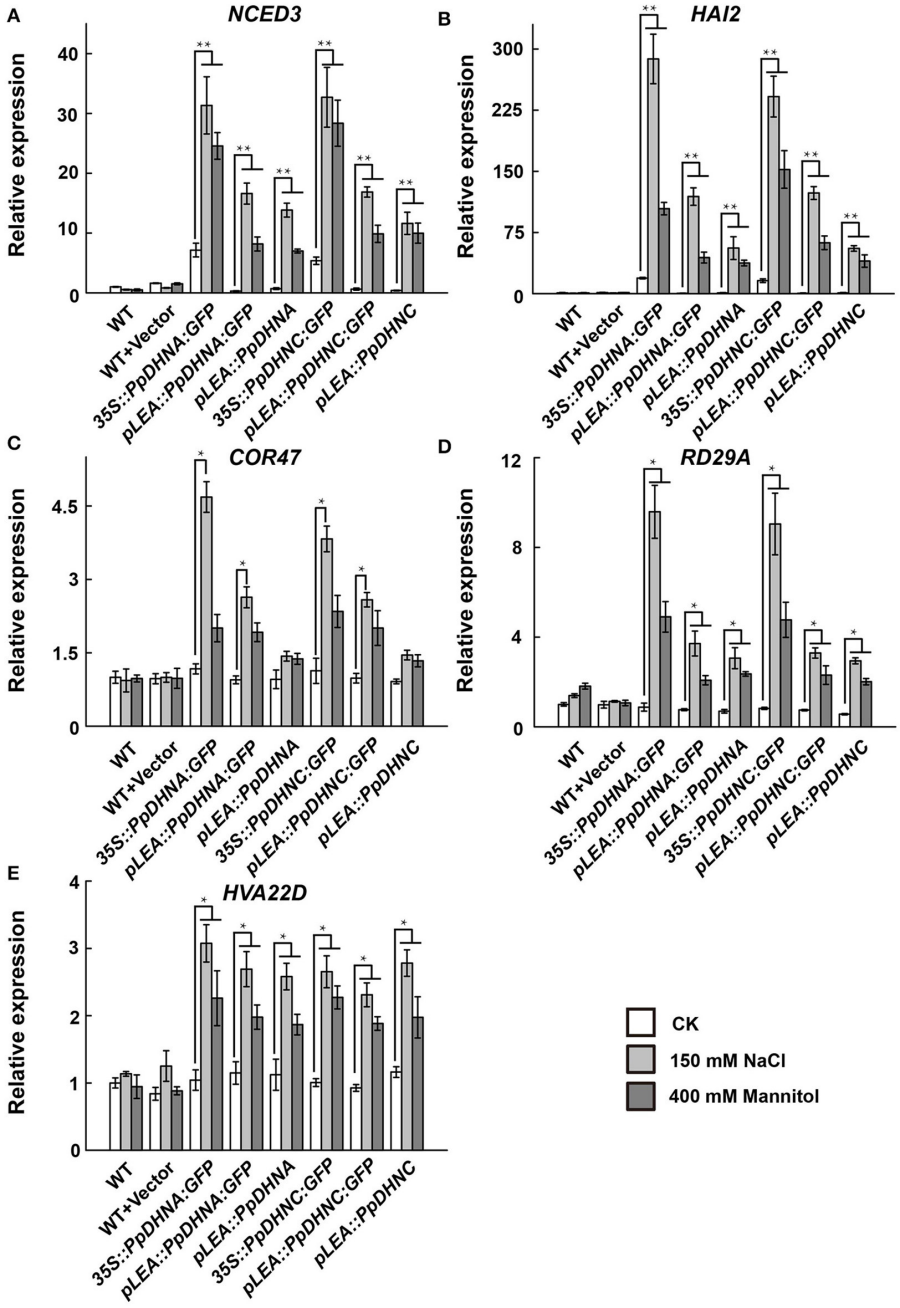
Expression analysis of stress-responsive genes in PpDHNA and PpDHNC transgentic lines and WT under salinity and osmotic stresses. **(A–E)** The expression of five stress-responsive genes (NCED3, HAI2, COR47, RD29A, and HVA22D) in PpDHNA and PpDHNC transgenic lines and WT was analyzed by quantitative real-time PCR. Seedlings treated as in Figure 3A with 150 mM sodium chloride or 400 mM Mannitol for 24 h. Data are mean values ±SD, with three biological replicates for each sample. Data are statistically analyzed with one-way ANOVA (LSD and Tamhane). Asterisks indicate significant different (^*^P < 0.05; ^**^P < 0.01).

To further examine the molecular mechanism underlying the salt and osmotic tolerance conferred by PpDHN, the expression levels of five stress-responsive marker genes in PpDHN transgenic lines and WT plants under treatment with 400 mM mannitol and 150 mM NaCl were examined by qRT-PCR (Figure 5). The five stress-responsive marker genes examined showed significantly upregulated transcription in WT and transgenic plants under conditions of stress. Moreover, the fold changes in upregulation of the five marker genes under each treatment were much higher in PpDHN transgenic lines, especially in PpDHN-overexpressing lines, compared to WT controls.

### PpDHNA and PpDHNC plants resist oxidative damage by increasing ROS scavenger accumulation

**Figure 6:**
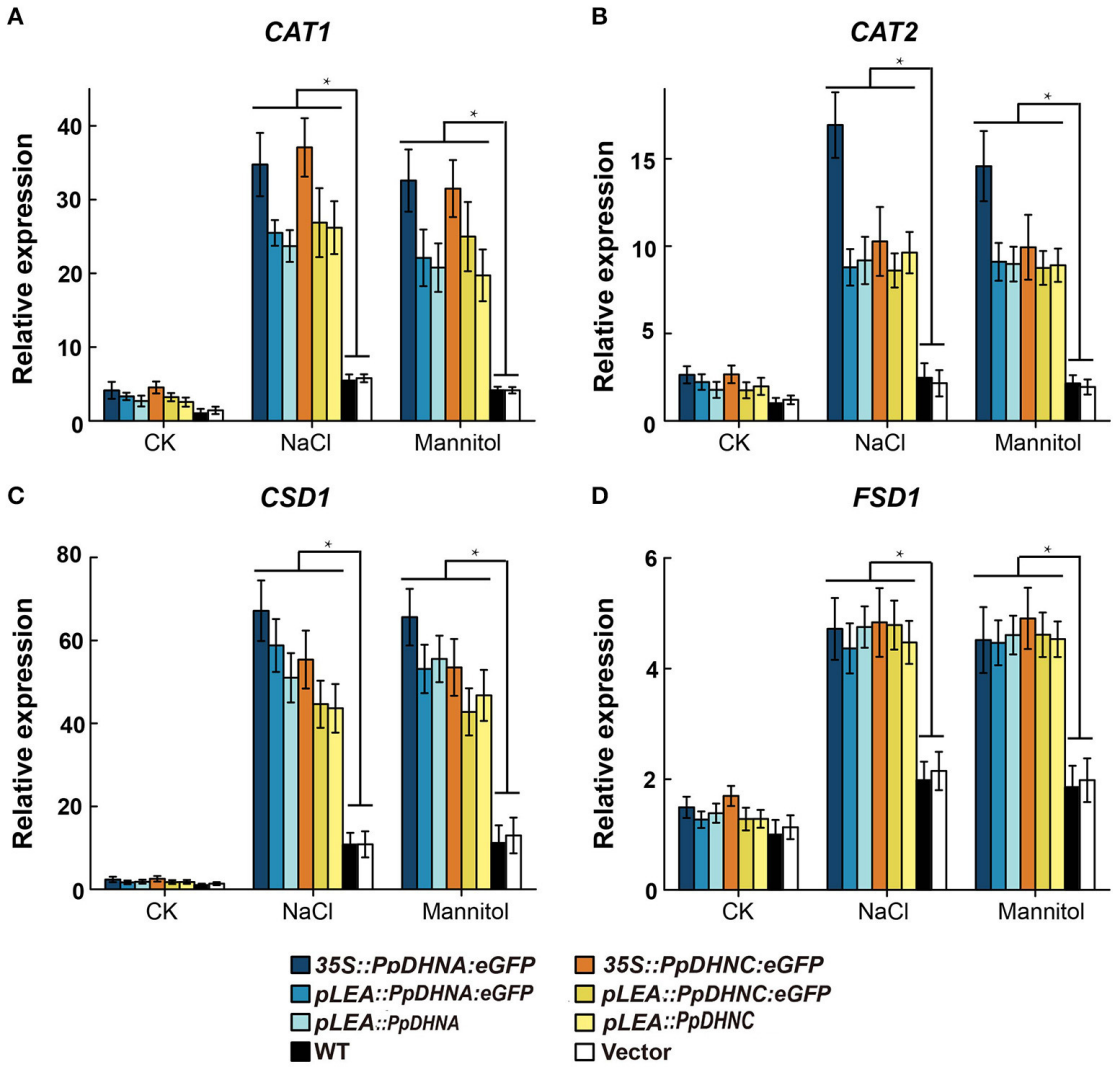
Expression analysis of ROS scavenger-related genes in PpDHNA and PpDHNC transgentic lines and WT under salinity and osmotic stresses. **(A–D)** The expression of four ROS scavenger-related genes (CAT1, CAT2, CSD1, and FSD1) in PpDHNA and PpDHNC transgenic lines and WT was analyzed by quantitative real-time PCR. Seedlings treated as in Figure 3A with 150 mM sodium chloride or 400 mM Mannitol for 24 h. Data are mean values ±SD, with three biological replicates for each sample. Data are statistically analyzed with one-way ANOVA (LSD and Tamhane). Asterisks indicate significant different (^*^P < 0.05).

Abiotic stresses, such as drought and high salinity, can induce the generation of reactive oxygen species (ROS), leading to oxidative damage to plants (Miller et al., 2010; Krasensky and Jonak, 2012). To examine the molecular mechanism of ROS scavenger production underlying the osmotic and salt tolerance conferred by PpDHN, we examined the expression of four ROS scavenger-related genes in PpDHN transgenic lines and WT controls under conditions of treatment with 400 mM mannitol and 150 mM NaCl (Figure 6). The four ROS scavenger-related genes examined were significantly upregulated in WT and transgenic plants under conditions of stress. However, the fold changes in ROS scavenger-related gene upregulation under each treatment condition were much higher in the PpDHN transgenic lines, especially the PpDHN-overexpressing lines, compared to in the WT controls. We speculate that the ROS scavenger-related enzyme activity should be higher in PpDHN transgenic plants.

**Figure 7:**
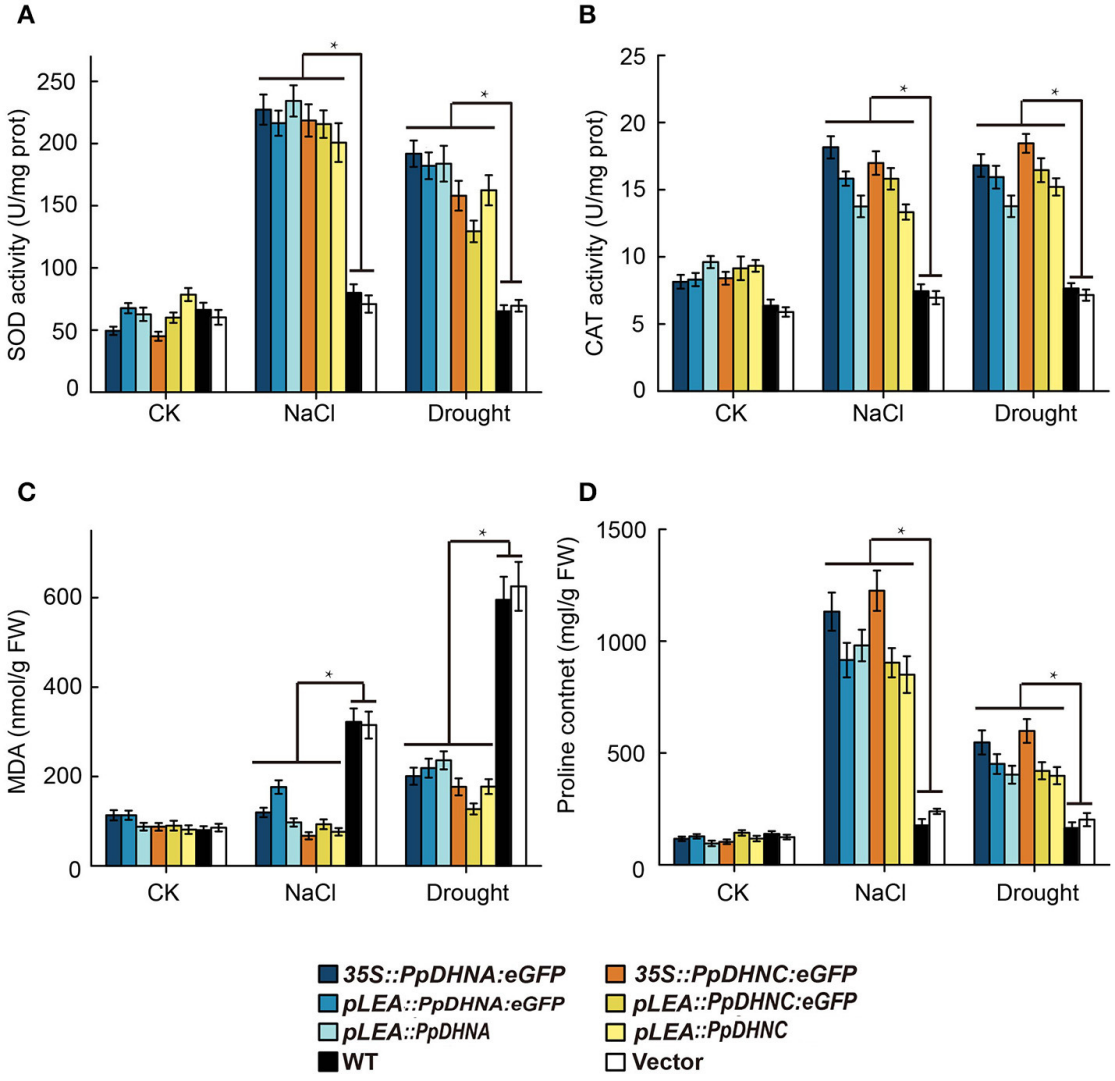
Analysis of oxidative scavenging and oxidative damage on the PpDHNA and PpDHNC transgenic Arabidopsis plants. **(A,B)** SOD and CAT activity of transgenic plants treated as in Figure 4A. Data are means of analysis from 0.05 g rosette leaves or cauline leaves ± SD, with three replicates for each sample. **(C,D)** MDA and Proline contents of transgenic plants treated as in Figure 4A. Data are means of analysis from 0.05 g rosette leaves or cauline leaves ±SD, with three replicates for each sample. Data are statistically analyzed with one-way ANOVA (LSD and Tamhane).

ROS scavenger analysis of DHN transgenic plants showed that the PpDHN protein conferred stronger stress tolerance to plants due to upregulation of SOD and CAT enzyme activities (Figures 7A,B). These changes result in clearance of excessive ROS accumulated under conditions of stress. MDA is considered an important indicator of plant oxidative stress, a sound membrane structure, and lipid peroxidation in response to salinity and drought (Xu et al., 2010; Wen et al., 2012). In the same way, the accumulation of proline reflects, to a large extent, the plant antioxidant ability (Matysik et al., 2002). MDA and proline contents reflect the ability of plants to resist oxidative damage (Figures 7C,D). The results indicated that PpDHN transgenic plants showed a strong antioxidant capacity. Thus, PpDHN was shown to enhance the ability of plants to resist stress by participating in the antioxidant pathway.

## Discussion

Environmental stress is an important cause of crop reduction. A great deal of research has focused on the genes induced by stress to elucidate the genetic and molecular bases of stress tolerance in plants. Various genes were shown to be upregulated in response to environmental stress (Zhu et al., 1997; Oono et al., 2003). Here, we focused on the DHNs to explore the mechanism of plant response to stress. Under conditions of cell dehydration, this type of protein often accumulates at high levels and is suggested to play a role in abiotic stress tolerance, although the mechanisms remain unclear.

A considerable number of studies have indicated that DHNs protect plants from stress injury by it's special molecular structure and disordered state (Bokor et al., 2005; Mouillon et al., 2008; Cuevas-Velazquez et al., 2014). Sequence analysis indicated that PpDHN is highly hydrophilic (Figure S1) and possesses a number of specific domains (Figures 1A–C). These properties are regarded as a key factors in reducing water loss in the plant under conditions of stress; this is equivalent to the role of the hydration buffers in maintaining water balance (Roychoudhury and Nayek, 2014).

The maintenance of membrane stability and integrity is of the highest priority for the maintenance of intracellular osmotic pressure. MDA is an indicator of membrane damage (Taulavuori et al., 2001), and it was accumulated to much lower levels in PpDHN plants compared to WT controls under conditions of drought and salt stress (Figure 7C), suggesting that PpDHN improved membrane integrity under stress. This protective effect was attributed to the disordered structure of DHN (Saibi et al., 2015a), meaning that it can easily bind other macromolecules, such as proteins, nucleic acids, and negatively charged lipids (Campbell and Close, 1997; Waterer et al., 2010; Ferrie et al., 2011). The fusion protein 35S::PpDHNC::GFP was mainly concentrated in the chloroplast (Figure 2). This specific chloroplast localization suggests that PpDHNC could protect the stability of the thylakoid and chloroplast membranes, and thus maintain electron transfer chain transmission in the photosystem. This makes it possible to carry out normal photosynthesis and alleviate the damage to the electron transport chain induced by stress (Ali et al., 2006; Asada, 2006; Fischer et al., 2013).

DHN proteins accumulate in vegetative tissues that have been exposed to multiple types of stress (Bray, 1994; Biesethève et al., 2008; Hundertmark and Hincha, 2008). The expression level of DHN protein and plant stress tolerance showed a positive correlation (Figure S6) (Yoon et al., 2009). Compared with plants with normal or low levels of DHNs, those expressing elevated levels of DHN show excellent stress tolerance (Puhakainen et al., 2004; Saavedra et al., 2006). In different species, heterologous expression of DHNs confers stress tolerance to plants (Xu et al., 1996; Cheng et al., 2002; Hara et al., 2003; Brini et al., 2010). Therefore, there is a great deal of interest in screening to identify the DHN genes conferring the greatest degrees of stress tolerance as targets for genetic engineering. The growth of seedlings and the phenotypic analysis of plants in soil showed that the PpDHN transgenic plants, especially overexpressing lines, were highly resistant to stress (Figures 3A, 4A). PpDHN-overexpressing lines showed significant root elongation and appropriate chlorophyll contents and also showed a phenotype similar to those reported in previous studies (Brini et al., 2007; Miller et al., 2010).

There is accumulating evidence that DHN transgenic plants show good survival, including outstanding growth and reduced injury, under various conditions of stress. However, there have been few reports regarding the mechanism of stress tolerance. The enhanced stress tolerance is mainly attributed to the upregulation of stress-responsive genes, such as *NCED3, HAI2, COR47, RD29A, and HVA22D* (Xue et al., 2011; Mao et al., 2012, 2014; Chu et al., 2015; Huang et al., 2015; Jia et al., 2015). The expression of these genes alters the physiological and metabolic processes of plants, thus conferring resistance to abiotic stress (Guo et al., 1992; Lin and Thomashow, 1992; Kiyosue et al., 1993; Msanne et al., 2011). Here, we performed expression level analysis of the above-mentioned genes and found positive correlations between the accumulation of PpDHN protein and the expression levels of these stress-responsive genes in the mutants, especially in PpDHN-overexpressing lines (Figure 5). Remarkably, some stress-responsive genes in overexpressed lines were also upregulated under normal conditions. This was likely because the PpDHN was derived from *P. patens*, which can survive in severe environments (Frank et al., 2005; Saavedra et al., 2006; Charron and Quatrano, 2009; Wang et al., 2009). Thus, PpDHN preadapted plants to the advent of stress. Our data indicated that PpDHNs increased the levels of expression of stress-related genes resulting in the protection of plants from abiotic stress.

The damage caused by abiotic stress is mainly reflected in the excessive accumulation of ROS, thus leading to oxidative damage (Gill and Tuteja, 2010). ROS are believed to be signaling molecules regulating plant growth and responses to biotic or abiotic stresses (Apel and Hirt, 2004; Mittler et al., 2004). DHNs enhanced plant oxidative damage tolerance by improving ROS scavenging ability, mainly reflected in the increases in the contents of ROS scavengers, such as SOD and CAT (Bowler et al., 1994; Mhamdi et al., 2010). We found that the expression of ROS scavenger synthesis-related genes, such as *CAT1, CAT2, CSD1*, and *FSD1* (Xie et al., 2012), were upregulated in PpDHN-overexpressing plants under conditions of drought and salt stress (Figures 6A–D). Elevated levels of SOD and CAT enzyme activities were detected accompanying the trends in expression of these genes (Figures 7A,B). Additionally, PpDHN transgenic plants accumulated higher levels of proline (Figure 7D), which allowed the plants to recover after removal of the stressor (Ben Rejeb et al., 2014). This was consistent with previous reports that DHN transgenic plants showed enhanced stress tolerance via improved ROS scavenging capacity (Saibi et al., 2015b).

In summary, the results of this study indicated that PpDHNs function as positive response proteins under conditions of drought and salt stress. PpDHN transgenic plants showed stronger tolerance to drought and salt stresses as determined by root elongation at the seedling or growth stage and by chlorophyll content while in the soil. Moreover, the results indicated that the enhanced stress tolerance of PpDHN transgenic plants was attributable to enhanced ROS scavenging capability and preservation of the integrity of the membrane structure and function. This makes sense from an evolutionary viewpoint, as a great variety of DHN types and functions have developed in the process of species evolution. The functions of homologous proteins in the same family can be determined by heterologous expression of DHNs in different species. Such studies are also important for the selection of DHN transgenic plants to increase agricultural production.

## Materials and methods

### Plasmid constructions and plant transformation

Vector PZP111 (Hajdukiewicz et al., 1994) was used to construct PZP111-eGFP. NOS-terminator (between EcoR1 and SacI restriction sites) and the eGFP fragment (start code ATG artificially mutated to ATC) between KpnI and SacI restriction sites were obtained from vector PBI121 and then recombined into vector PZP111, respectively. The resultant vector was named PZP111-eGFP. 35S CaMV promoter from PBI121 (with HindIII and XbaI) was recombined into vector PZP111-eGFP to obtain vector PZP111-35SeGFP (Figure S7).

Specific primer pair (*pLEA-PstI-F* and *pLEA-SalI-R*, Table S2), was designed upstream of the open reading frame to amplify the PpDHNA promoter. The length of the cloned upstream fragment flanked by PstI and SalI restriction sites, as shown by the underlining. A pair of gene-specific primers (*PpDHNA-XbaI-F* and *PpDHNA- BamHI-R*, Table S2) was designed to amplify the full length open reading frame of PpDHNA. The CDS without the stop codon and flanked by XbaI and BamHI enzyme sites, was amplified from cDNA. The CDS fragment was cloned into pPZP111-35S- eGFP to obtain 35S::*PpDHNA*::eGFP.

The native promoter and the CDS of the gene were cloned into pPZP111-eGFP to obtain pLEA::*PpDHNA*::GFP. To obtain pLEA::*PpDHNA*, the full gene CDS was amplified using *PpDHNA-SalI-F* and *PpDHNA-BamHI-R* (Table S2). The same strategy was adopted to construct pLEA::*PpDHNC*, pLEA::*PpDHNC*::GFP, and 35S::*PpDHNC*::GFP (Information of primers used are listed in Table S2).

Transgenic lines were generated using the Agrobacterium tumefaciens LBA4404 vacuum infiltration method. This method is usually done by stigma infiltration. Transgenic plants were obtained by screening progeny. Seeds of the first-generation transgenic line T1 from infiltrated plants were germinated on 1/2 MS medium containing 50 mg L^−1^ kanamycin to select for the positive seedlings. Several lines were obtained for each transformation and at least two generations of resistance screening were performed (copy number in Figure S5 and Table S4, Figure S8 shows PCR analysis of the transgenic plants).

### Plant materials cultivation and physiological analysis of stress treatments

All of the Arabidopsis seeds and transgenic plants used in this study were in a Col-0 (Columbia) background. All plants were grown in a controlled growth chamber at 21–22°C under cool-white fluorescent light (80–100 μmol m^−2^ s^−1^) in a long day photoperiod (16 h light/8 h dark).

Arabidopsis seeds were germinated on half-strength Murashige and Skoog (1/2 MS) medium supplemented with 0.6% (w/v) sucrose and 0.7% (w/v) agar. After 7 days, seedlings were transferred to 1/2 MS medium with 100–400 mM mannitol or 50–150 mM NaCl and treated for another 7 days. Mannitol and NaCl were added to different 1/2 MS medium. Seven-day seedlings(normal 1/2 MS medium) were transferred to above-mentioned 1/2 MS medium.

Arabidopsis were also grown in garden soil (Basic substrate No. 1, Pindstrup Mosebrug A/S, Denmark) without additional fertilizer, and were maintained in a greenhouse under standard growing conditions (Weigel and Glazebrook, 2002). Before salinity and drought treatment, young plants were watered once per week. Three-week plants were used for salinity or drought treatment, by watering with 200 mM NaCl solution or depriving of water respectively for 4 weeks. After drought treatment, they are rewaterd for a week.

### Measurements of chlorophyll, MDA and proline contents

Chlorophyll content was measured as previously reported (Wolken and Schwertz, 1953). Rosette leaves were taken and weighed at the indicated time, then placed into 5 mL of 90% (v/v) acetone for extraction of chlorophyll a/b. The chlorophyll content of each sample was assayed by measuring the absorbance at 645, 663, and 652 nm using a Beckman DU-640 spectrophotometer.

$$\begin{array}{l} \text{chlorophyl a }(\text{Chla})(\text{mg/g})=(12.7\times \text{A663}-2.697\times \text{A645})\\ \;\times \;7\times \text{V}/(1000\times \text{W})\\ \text{chlorophyll b }(\text{Chlb})(\text{mg/g})=(22.77\times \text{A645}-4.687\times A663)\\ \;\times \;7\times \text{V}/(1000\times \text{W})\\ \text{chlorophyll }(\text{Chl})(\text{mg/g})=\text{chlorophyll a }(\text{Chla})\\ \;+\text{ chlorophyll b }(\text{Chlb})(\text{mg/g}) \end{array}$$

(V:The final volume of extraction solution) (W: Leaf fresh weight)

Measurement of MDA: 0.05 g of rosette leaves or cauline leaves was ground in liquid nitrogen. Each sample was incubated with 5 ml of 10% (w/v) trichloroacetic acid (TCA) solution, then with the same volume 0.6% (w/v) of 2-thiobarbituric acid (TBA) buffer. The MDA content of each sample was assayed by measuring the absorbance at 450, 532, and 600 nm using a Beckman DU-640 spectrophotometer. MDA content were calculated with the following formula:

$$\begin{array}{l} \text{MDA levels }(\text{nmol }{\text{g}}^{-1}\text{ FW})=[6.452\times (\text{D532}-\text{D600})-0.559\\ \;\times \text{ D450}]\times 50 \end{array}$$

Proline content was measured using the colorimetric determination based on proline's reaction with ninhydrin (Bates et al., 1973). 0.05 g of rosette leaves or cauline leaves were incubated with 2 mL of 3% sulfosalicylic acid solution and boiled for 30 min. The proline solution was mixed with the same volume of ninhydrin acid and glacial acid at 100°C for 30 min. After mixture had cooled down, the proline content of each sample was assayed by measuring the absorbance at 520 nm using a Beckman DU-640 spectrophotometer.

### Measurement of plant water potential

Leaf water potential (ψ) was estimated on rosette leaves after drought or salt treatment. 0.5 g leaves in each plot were used to determine water potential using a DECAGON WP4C pressure chamber.

### Oxidative enzyme assays

The antioxidant enzyme activities of superoxide dismutase (SOD), and catalase (CAT) were determined by ELISA using the detection kits following the manufacturer's instructions (Jiancheng Bioengineering Institute, Nanjing, China).

### Sequence databases, alignment, and phylogeny

BLASTp was used to search for homologs of PpDHNA and PpDHNC proteins in the complete sequenced genomes of plants from various biological databases (GenBank, protein database, and genomes database). Sequences from several species were aligned with ClustalW, and phylogenetic tree was constructed using the BioNJ software (Gascuel, 1997) with Maximum likehood method (ML) analysis.

### Microscopy

Samples were collected from the 2nd true leaves of 7-day-old seedlings. Leaf tips were fixed in 3.5% glutaraldehyde for 1 h (dark; room temperature, RT), then incubated in 0.1 M Na2EDTA (pH 9.0) solution for additional 2 h in dark at RT (Pyke and Leech, 1991). After that, leaf samples were transferred into 1 μg/mL DAPI (Sigma) solution, stained in dark for 20 min and washed with PBS buffer for 5 times before imaging.

GFP signal was examined using a Zeiss LSM 780 laser confocal microscope (Carl Zeiss, Germany). The excitation wavelength used was 488 nm. Emission wavelengths between 503 and 518 nm were used to detect GFP and wavelengths between 590 and 608 nm were used to detect chlorophyll auto-fluorescence. The excitation wavelength and emission wavelengths used for DAPI imaging are 405 nm and between 446 and 475 nm respectively. The obtained images were subsequently analyzed using Adobe Photoshop CS5.1 software.

### Isolation of RNA, cDNA synthesis, and quantitative real-time PCR

Total RNA was isolated using Trizol reagent (Invitrogen, Gaithersburg, MD, USA) according to manufacturer's instructions. PrimeScript™ RT Master Mix (Perfect Real Time) was used for RNA purification and reverse transcription following the manufacturer's instructions. Real-time quantitative reverse transcription-PCRs (RT-PCRs) were performed using a QuantStudio™ 6 Flex Real- Time PCR System (Applied Biosystems, Warrington, UK) with SYBR Pre-mix Ex TaqTM (TaKaRa Bio Inc., China) according to the manufacturer's instructions. Using specific primers (Table S3), the expression levels of the genes are presented as values relative to the corresponding control samples at the indicated times or under the indicated conditions after normalization to actin2 (Du et al., 2014) transcript levels.

## Author contributions

YHu and YHe designed the research. QLi, XZ, DZ, TQ, and YX performed research. YHu, QLi, and XZ analyzed data. QLi wrote the paper. QLv and FB revised the paper.

### Conflict of interest statement

The authors declare that the research was conducted in the absence of any commercial or financial relationships that could be construed as a potential conflict of interest. The reviewer RJ and handling Editor declared their shared affiliation, and the handling Editor states that the process met the standards of a fair and objective review.

## Supplementary material

Figure S1 Kyte and Doolittle hydropathy analysis of PpDHNA and PpDHNC. Values below the zero lines are negative and hydrophilic, based on the average over a moving window of 19 amino acids. The numbers on the horizontal axis refer to the amino acid positions.

Figure S2 Unrooted dendrogram of all Arabidopsis LEA genes and PpDHNA and PpDHNC. Sequence alignments were performed unsing the ClustalW algorithm and an unrooted dendrogram was drawn subsequently. The different LEA groups are indicated by different colors.

Figure S3 Salinity and osmotic stresses induced the transcriptional levels of PpDHNA and PpDHNC in transgenic plants. **(A,B)** Salinity-induced and osmotic-induced expression of PpDHNA and PpDHNC in the transgenic plants, analyzed using quantitative real-time PCR. Seedlings treated as in Figure 3A with 150 mM sodium chloride or 400 mM Mannitol for 24 h. Data are mean values ±SD, with three biological replicates for each sample. Data are statistically analyzed with one-way ANOVA (LSD and Tamhane). Asterisks indicate significant different (^*^*P* < 0.05; ^**^*P* < 0.01; ^***^*P* < 0.001).

Figure S4 Effects of salinity and osmotic stresses on the others lines of PpDHNA and PpDHNC transgenic Arabidopsis seedlings. **(A)** Phenotypic comparison of wild type and PpDHN transgenic seedlings treated as indicated for 7 days. **(B,C)** Salinity-induced and osmotic-induced expression of PpDHNA and PpDHNC in the transgenic plants, analyzed using quantitative real-time PCR. Seedlings treated as in **(A)** with 150 mM sodium chloride or 400 mM Mannitol for 24 h.

Figure S5 QRT-PCR analysis for copy number of genes. To plot standard curves, single copy vector was diluted to different concentrations. So did transgenic plants. By comparing the slope, each lines has a copy number similar to that of single copy vectors'.

Figure S6 PpDHNA and PpDHNC protein expression under stresses. Vector and PpDHN transgenic seedlings treated as Figure 3 for 7 days. Salinity-induced and osmotic-induced expression of PpDHNA and PpDHNC proteins in the transgenic plants, analyzed using western blot. Because other lines suffer severe stress, they cannot grow normally. The accumulated protein could not be detected. Therefore, only the expression of *35S::PpDHNA::GFP* and *35S::PpDHNC::GFP* transgenic lines were shown.

Figure S7 Gene constructs prepared in different vectors.

Figure S8 PCR analysis of the transgenic plants.

Table S1 Characteristics of genes encoding LEA proteins in *Arabidopsis thaliana* and PpDHNA and PpDHNC.

Table S2 The sequences of PCR primers for cloning.

Table S3 The sequences of PCR primers for real-time PCR.

Table S4 Copy numbers of different genes in disfferent transgenic plants.

## References

[B1] Afrasyab R. Richarda J. Kazem P. Rana M. (2010). Stomatal conductance as a screen for osmotic stress tolerance in durum wheat growing in saline soil. Funct. Plant Biol. 37, 255–263. 10.1071/FP09148

[B2] Ali A. Alqurainy F. Motohashi N. (2006). Activities of Antioxidants in Plants under Environmental Stress. Department of Botany and Microbiology, Faculty of Science, King Saud University, Riyadh.

[B2a] Agarwal T. Upadhyaya G. Halder T. Mukherjee A. Majumder A. L. Ray S. (2016). Different dehydrins perform separate functions in physcomitrella patens. Planta 245, 1–18. 10.1007/s00425-016-2596-1 27638172

[B3] Apel K. Hirt H. (2004). Reactive oxygen species: metabolism, oxidative stress, and signal transduction. Plant Biol. 55, 373–399. 10.1146/annurev.arplant.55.031903.141701 15377225

[B4] Artus N. N. Uemura M. Steponkus P. L. Gilmour S. J. Lin C. Thomashow M. F. (1996). Constitutive expression of the cold-regulated *Arabidopsis thaliana* cor15a gene affects both chloroplast and protoplast freezing tolerance. Proc. Natl. Acad. Sci. U.S.A. 93, 13404–13409. 10.1073/pnas.93.23.13404 11038526 PMC24106

[B5] Asada K. (2006). Production and scavenging of reactive oxygen species in chloroplasts and their functions 1. Plant Physiol. 141, 391–396. 10.1104/pp.106.082040 16760493 PMC1475469

[B6] Bates L. S. Waldren R. P. Teare I. D. (1973). Rapid determination of free proline for water-stress studies. Plant Soil 39, 205−207 10.1007/bf00018060

[B7] Battaglia M. (2008). The enigmatic lea proteins and other hydrophilins. Plant Physiol. 148, 6–24. 10.1104/pp.108.120725 18772351 PMC2528095

[B71] Ben Rejeb K. Abdelly C. Savouré A. (2014). How reactive oxygen species and proline face stress together. Plant Physiol. Biochem. 80, 278–284. 10.1016/j.plaphy.2014.04.007 24813727

[B8] Biesethève N. Gaubiercomella P. Debures A. Lasserre E. Jobet E. Raynal M. (2008). Inventory, evolution and expression profiling diversity of the lea (late embryogenesis abundant) protein gene family in *Arabidopsis thaliana*. Plant Mol. Biol. 67, 107–124. 10.1007/s11103-008-9304-x 18265943

[B9] Bokor M. Csizmok V. Kovacs D. Banki P. Friedrich P. Tompa P.. (2005). NMR relaxation studies on the hydrate layer of intrinsically unstructured proteins. Biophys. J. 88, 2030–2037. 10.1529/biophysj.104.051912 15613629 PMC1305255

[B10] Borovskii G. B. Stupnikova I. V. Antipina A. I. Vladimirova S. V. Voinikov V. K. V. K. (2002). Accumulation of dehydrin-like proteins in the mitochondria of cereals in response to cold, freezing, drought and aba treatment. BMC Plant Biol. 2:5. 10.1186/1471-2229-2-1 12057012 PMC116594

[B11] Bowler C. Van Camp W. Van Montagu M. Inze 'D. Asada K. (1994). Superoxide dismutase in plants. Crit. Rev. Plant Sci. 13, 199–218. 10.1080/07352689409701914

[B12] Bray E. A. (1994). Molecular responses to water deficit. Plant Physiol. 103, 1035–1040. 10.1104/pp.103.4.1035 12231998 PMC159086

[B13] Brini F. Hanin M. Lumbreras V. Amara I. Khoudi H. Hassairi A.. (2007). Overexpression of wheat dehydrin dhn-5 enhances tolerance to salt and osmotic stress in *Arabidopsis thaliana*. Plant Cell Reports 26, 2017–2026. 10.1007/s00299-007-0412-x 17641860

[B14] Brini F. Saibi W. Amara I. Gargouri A. Masmoudi K. Hanin M. (2010). Wheat dehydrin dhn-5 exerts a heat-protective effect on beta-glucosidase and glucose oxidase activities. Agric. Biol. Chem. 74, 1050–1054. 10.1271/bbb.90949 20460710

[B15] Campbell S. A. Close T. J. (1997). Dehydrins: genes, proteins, and associations with phenotypic traits. New Phytol. 137, 61–74. 10.1046/j.1469-8137.1997.00831.x

[B16] Charron A. J. Quatrano R. S. (2009). Between a rock and a dry place: the water- stressed moss. Mol. Plant 2, 478–486. 10.1093/mp/ssp018 19825631

[B17] Cheng Z. Targolli J. Huang X. Wu R. (2002). Wheat lea genes, pma80 and pma1959, enhance dehydration tolerance of transgenic rice (*Oryza sativa* l.). Mol. Breed. 10, 71–82. 10.1023/A:1020329401191

[B18] Choi D. W. Zhu B. Close T. J. (1999). The barley (hordeum vulgare l.) dehydrin multigene family: sequences, allele types, chromosome assignments, and expression characteristics of 11 dhn genes of cv dicktoo. Theor. Appl. Genet. 98, 1234–1247. 10.1007/s001220051189

[B19] Chu X. Wang C. Chen X. Lu W. Li H. Wang X.. (2015). The cotton WRKY gene GhWRKY41 positively regulates salt and drought stress tolerance in transgenic Nicotiana benthamiana. PLoS ONE 10:e0143022. 10.1371/journal.pone.0143022 26562293 PMC4643055

[B20] Close T. J. (1996). Dehydrins: emergence of a biochemical role of a family of plant dehydration proteins. Physiol. Plant. 97, 795–803. 10.1111/j.1399-3054.1996.tb00546.x

[B21] Close T. J. (1997). Dehydrins: a commonalty in the response of plants to dehydration and low temperature. Physiol. Plant. 100, 291–296. 10.1111/j.1399-3054.1997.tb04785.x

[B22] Close T. J. Kortt A. A. Chandler P. M. (1989). A cDNA-based comparison of dehydration-induced proteins (dehydrins) in barley and corn. Plant Mol. Biol. 13, 95–108. 10.1007/BF00027338 2562763

[B23] Cuevas-Velazquez C. L. Rendón-Luna D. F. Covarrubias A. A. (2014). Dissecting the cryoprotection mechanisms for dehydrins. Front. Plant Sci. 5:583. 10.3389/fpls.2014.00583 25400649 PMC4212605

[B24] Du J. Li M. Kong D. Wang L. Lv Q. Wang J.. (2014). Nitric oxide induces cotyledon senescence involving co-operation of the NES1/MAD1 and EIN2-associated ORE1 signalling pathways in Arabidopsis. J. Exp. Bot. 65, 4051–4063. 10.1093/jxb/ert429 24336389 PMC4106434

[B25] Dure L. Crouch M. Harada J. Ho T. H. D. Mundy J. Quatrano R.. (1989). Common amino acid sequence domains among the lea proteins of higher plants. Plant Mol. Biol. 12, 475–486. 10.1007/BF00036962 24271064

[B26] Eriksson S. K. Kutzer M. Procek J. Gröbner G. Harryson P. (2011). Tunable membrane binding of the intrinsically disordered dehydrin lti30, a cold-induced plant stress protein. Plant Cell 23, 2391–2404. 10.1105/tpc.111.085183 21665998 PMC3160030

[B27] Ferrie A. M. R. Bethune T. D. Arganosa G. C. Waterer D. (2011). Field evaluation of doubled haploid plants in the apiaceae: dill (*Anethum graveolens*, l.), caraway (*Carum carvi*, l.), and fennel (Foeniculum vulgare, mill.). Plant Cell Tiss. Organ Cult. 104, 407–413. 10.1007/s11240-010-9821-6

[B28] Fischer B. B. Hideg É. Krieger-Liszkay A. (2013). Production, detection, and signaling of singlet oxygen in photosynthetic organisms. Antioxid. Redox Signal. 18, 2145–2162. 10.1089/ars.2012.5124 23320833

[B29] Frank W. Ratnadewi D. Reski R. (2005). Physcomitrella patens, is highly tolerant against drought, salt and osmotic stress. Planta 220, 384–394. 10.1007/s00425-004-1351-1 15322883

[B30] Garayarroyo A. Colmeneroflores J. M. Garciarrubio A. Covarrubias A. A. (2000). Highly hydrophilic proteins in prokaryotes and eukaryotes are common during conditions of water deficit. J. Biol. Chem. 275, 5668–5674. 10.1074/jbc.275.8.5668 10681550

[B31] Gascuel O. (1997). BIONJ: an improved version of the NJ algorithm based on a simple model of sequence data. Mol. Biol. Evol. 14, 685–695. 9254330 10.1093/oxfordjournals.molbev.a025808

[B32] Gill S. S. Tuteja N. (2010). Reactive oxygen species and antioxidant machinery in abiotic stress tolerance in crop plants. Plant Physiol. Biochem. 48, 909–930. 10.1016/j.plaphy.2010.08.016 20870416

[B33] Grelet J. (2004). Identification et Caractérisation Moléculaire d'une Protéine LEA (Late Embryogenesis Abundant) Mitochondriale Exprimée dans les Semences de Pois. Doctoral dissertation, Angers.

[B34] Guo W. W. Ward R. W. Thomashow M. F. (1992). Characterization of a cold- regulated wheat gene related to Arabidopsis cor47. Plant Physiol. 100, 915–922. 10.1104/pp.100.2.915 16653076 PMC1075644

[B35] Hajdukiewicz P. Svab Z. Maliga P. (1994). The small, versatile ppzp family of agrobacterium binary vectors for plant transformation. Plant Mol. Biol. 25, 989–994. 10.1007/BF00014672 7919218

[B36] Hara M. Terashima S. Fukaya T. Kuboi T. (2003). Enhancement of cold tolerance and inhibition of lipid peroxidation by citrus dehydrin in transgenic tobacco. Planta 217, 290–298. 10.1007/s00425-003-0986-7 12783337

[B37] Heyen B. J. Alsheikh M. K. Smith E. A. Torvik C. F. Seals D. F. Randall S. K. (2002). The calcium-binding activity of a vacuole-associated, dehydrin-like protein is regulated by phosphorylation. Plant Physiol. 130, 675–687. 10.1104/pp.002550 12376635 PMC166597

[B38] Houde M. Dallaire S. N'Dong D. Sarhan F. (2004). Overexpression of the acidic dehydrin wcor410 improves freezing tolerance in transgenic strawberry leaves. Plant Biotechnol. J. 2, 381–387. 10.1111/j.1467-7652.2004.00082.x 17168885

[B39] Houde M. Dhindsa R. S. Sarhan F. (1992). A molecular marker to select for freezing tolerance in gramineae. Mol. Genet. Genomics 234, 43–48. 1495483 10.1007/BF00272343

[B40] Huang Q. Wang Y. Li B. Chang J. Chen M. Li K.. (2015). TaNAC29, a NAC transcription factor from wheat, enhances salt and drought tolerance in transgenic Arabidopsis. BMC Plant Biol. 15:268. 10.1186/s12870-015-0644-9 26536863 PMC4632686

[B41] Hundertmark M. Hincha D. K. (2008). Lea (late embryogenesis abundant) proteins and their encoding genes in *Arabidopsis thaliana*. BMC Genomics 9:118. 10.1186/1471-2164-9-118 18318901 PMC2292704

[B42] Jensen A. B. Goday A. Figueras M. Jessop A. C. Pagès M. (1998). Phosphorylation mediates the nuclear targeting of the maize rab17 protein. Plant J. Cell Mol. Biol. 13, 691–697. 10.1186/1471-2164-9-118 9681011

[B43] Jia H. Wang C. Wang F. Liu S. Li G. Guo X. (2015). GhWRKY68 reduces resistance to salt and drought in transgenic Nicotiana benthamiana. PLoS ONE 10:e0120646. 10.1371/journal.pone.0120646 25793865 PMC4368093

[B44] Karami A. Shahbazi M. Niknam V. Shobbar Z. S. Tafreshi R. S. Abedini R. (2013). Expression analysis of dehydrin multigene family across tolerant and susceptible barley (*Hordeum vulgare*, l.) genotypes in response to terminal drought stress. Acta Physiol. Plant. 35, 2289–2297. 10.1007/s11738-013-1266-1

[B45] Kiyosue T. Yamaguchi-Shinozaki K. Shinozaki K. (1993). Characterization of two cDNAs (ERD11 and ERD13) for dehydration-inducible genes that encode putative glutathione S-transferases in *Arabidopsis thaliana* L. FEBS Lett. 335, 189–192. 10.1016/0014-5793(93)80727-C 8253194

[B46] Koag M. C. Fenton R. D. Wilkens S. Close T. J. (2003). The binding of maize dhn1 to lipid vesicles. gain of structure and lipid specificity. Plant Physiol. 131, 309–316. 10.1104/pp.011171 12529538 PMC166810

[B47] Koag M. C. Wilkens S. Fenton R. D. Resnik J. Vo E. Close T. J. (2009). The k-segment of maize dhn1 mediates binding to anionic phospholipid vesicles and concomitant structural changes. Plant Physiol. 150, 1503–1514. 10.1104/pp.109.136697 19439573 PMC2705017

[B48] Krasensky J. Jonak C. (2012). Drought, salt, and temperature stress-induced metabolic rearrangements and regulatory networks. J. Exp. Bot. 63, 1593–1608. 10.1093/jxb/err460 22291134 PMC4359903

[B49] Liii D. (1993). Structural motifs in lea proteins. Curr. Top. Plant Physiol. 10, 91–103.

[B50] Lin C. Thomashow M. F. (1992). DNA sequence analysis of a complementary DNA for coldregulated Arabidopsis gene cor15 and characterization of the COR 15 polypeptide. Plant Physiol. 99, 519–525. 16668917 10.1104/pp.99.2.519 PMC1080494

[B51] Liu Y. Wang L. Xing X. Sun L. Pan J. Kong X.. (2013). Zmlea3, a multifunctional group 3 lea protein from maize (*Zea mays* l.), is involved in biotic and abiotic stresses. Plant Cell Physiol. 54, 944–959. 10.1093/pcp/pct047 23543751

[B52] Mao X. Chen S. Li A. Zhai C. Jing R. (2014). Novel NAC transcription factor TaNAC67 confers enhanced multi-abiotic stress tolerances in Arabidopsis. PLoS ONE 9:e84359. 10.1371/journal.pone.0084359 24427285 PMC3888409

[B53] Mao X. Zhang H. Qian X. Li A. Zhao G. Jing R. (2012). TaNAC2, a NAC-type wheat transcription factor conferring enhanced multiple abiotic stress tolerances in Arabidopsis. J. Exp. Bot. 63, 2933–2946. 10.1093/jxb/err462 22330896 PMC3350912

[B54] Matysik J. Bhalu B. A. Mohanty P. Bohrweg N. (2002). Molecular mechanisms of quenching of reactive oxygen species by proline under stress in plant. Curr Sci. 82, 525–532.

[B55] Mhamdi A. Queval G. Chaouch S. Vanderauwera S. Breusegem F. V. Noctor G. (2010). Catalase function in plants: a focus on arabidopsis mutants as stress-mimic models. J. Exp. Bot. 61, 4197–4220. 10.1093/jxb/erq282 20876333

[B56] Miller G. Suzuki N. Ciftci-Yilmaz S. Mittler R. (2010). Reactive oxygen species homeostasis and signalling during drought and salinity stresses. Plant Cell Environ. 33, 453–467. 10.1111/j.1365-3040.2009.02041.x 19712065

[B57] Mittler R. Vanderauwera S. Gollery M. Van Breusegem F. (2004). Reactive oxygen gene network of plants. Trends Plant Sci. 9, 490–498. 10.1016/j.tplants.2004.08.009 15465684

[B58] Mouillon J. M. Eriksson S. K. Harryson P. (2008). Mimicking the plant cell interior under water stress by macromolecular crowding: disordered dehydrin proteins are highly resistant to structural collapse. Plant Physiol. 148, 1925–1937. 10.1104/pp.108.124099 18849483 PMC2593683

[B59] Mouillon J. M. Gustafsson P. Harryson P. (2006). Structural investigation of disordered stress proteins. comparison of full-length dehydrins with isolated peptides of their conserved segments. Plant Physiol. 141, 638–650. 10.1104/pp.106.079848 16565295 PMC1475461

[B60] Msanne J. Lin J. Stone J. M. Awada T. (2011). Characterization of abiotic stress- responsive *Arabidopsis thaliana* RD29A and RD29B genes and evaluation of transgenes. Planta 234, 97–107. 10.1007/s00425-011-1387-y 21374086

[B61] Mueller J. K. Fernando D. (2003). Identification of a chloroplast dehydrin in leaves of mature plants. Int. J. Plant Sci. 164, 535–542. 10.1086/375376

[B62] Mundy J. Chua N. H. (1988). Abscisic acid and water-stress induce the expression of a novel rice gene. Embo J. 7, 2279–2286. 2973410 10.1002/j.1460-2075.1988.tb03070.x PMC457090

[B63] Munns R. Tester M. (2008). Mechanisms of salinity tolerance. Plant Biol. 59, 651–681. 10.1146/annurev.arplant.59.032607.092911 18444910

[B64] Neven L. G. Haskell D. W. Hofig A. Li Q. B. Guy C. L. (1993). Characterization of a spinach gene responsive to low temperature and water stress. Plant Mol. Biol. 21, 291–305. 10.1007/BF00019945 8425058

[B65] Nylander M. Svensson J. Palva T. P. Welin B. V. (2001). Stress-induced accumulation and tissue-specific localization of dehydrins in *Arabidopsis thaliana*. Plant Mol. Biol. 45, 263–279. 10.1023/A:1006469128280 11292073

[B66] Oono Y. Seki M. Nanjo T. Narusaka M. Fujita M. Satoh R.. (2003). Monitoring expression profiles of arabidopsis, gene expression during rehydration process after dehydration using ca. 7000 full-length cdna microarray. Plant J. 34, 868–887. 10.1046/j.1365-313X.2003.01774.x 12795706

[B67] Porat R. Pasentsis K. Rozentzvieg D. Gerasopoulos D. Falara V. Samach A.. (2004). Isolation of a dehydrin cdna from orange and grapefruit citrus fruit that is specifically induced by the combination of heat followed by chilling temperatures. Physiol. Plant. 120, 256–264. 10.1111/j.0031-9317.2004.0242.x 15032860

[B68] Puhakainen T. Hess M. W. Mäkelä P. Svensson J. Heino P. Palva E. T. (2004). Overexpression of multiple dehydrin genes enhances tolerance to freezing stress in arabidopsis. Plant Mol. Biol. 54, 743–753. 10.1023/B:PLAN.0000040903.66496.a4 15356392

[B69] Pyke K. A. Leech R. M. (1991). Rapid image analysis screening procedure for identifying chloroplast number mutants in mesophyll cells of *Arabidopsis thaliana* (L.) Heynh. Plant Physiol. 96, 1193−1195 10.1104/pp.96.4.1193 16668319 PMC1080914

[B70] Rahman L. N. Chen L. Nazim S. Bamm V. V. Yaish M. W. Moffatt B. A.. (2010). Interactions of intrinsically disordered thellungiella salsuginea dehydrins tsdhn-1 and tsdhn-2 with membranes - synergistic effects of lipid composition and temperature on secondary structure. Biochem. Cell Biol. 88, 791–807. 10.1139/O10-026 20921991

[B72] Roberts J. K. DeSimone N. A. Lingle W. L. Dure L. III. (1993). Cellular concentrations and uniformity of cell-type accumulation of two lea proteins in cotton embryos. Plant Cell 5, 769–780. 10.1105/tpc.5.7.769 12271086 PMC160315

[B73] Rorat T. Szabala B. M. Grygorowicz W. J. Wojtowicz B. Yin Z. Rey P. (2006). Expression of sk3-type dehydrin in transporting organs is associated with cold acclimation in solanum species. Planta 224, 205–221. 10.1007/s00425-005-0200-1 16404580

[B74] Roychoudhury A. Nayek S. (2014). Structural aspects and functional regulation of late embryogeniesis abundant (LEA) genes and proteins conferring abiotic stress tolerance in plants, in Abiotic Stress: Role in Sustainable Agriculture, Detrimental Effects and Management Strategies, ed Annabella F. (New York, NY: Nova Publishers), 43–109.

[B75] Rozema J. Flowers T. (2008). Ecology. crops for a salinized world. Science 322, 1478–1480. 10.1126/science.1168572 19056965

[B76] Saavedra L. Svensson J. Carballo V. Izmendi D. Welin B. Vidal S. (2006). A dehydrin gene in physcomitrella patens is required for salt and osmotic stress tolerance. Plant J. 45, 237–249. 10.1111/j.1365-313X.2005.02603.x 16367967

[B76a] Ruibal C. Salamó I. P. Carballo V. Castro A. Bentancor M. Borsani O.. (2012). Differential contribution of individual dehydrin genes from physcomitrella patens to salt and osmotic stress tolerance. Plant Sci. Int. J. Exp. Plant Biol. 190, 89–102. 10.1016/j.plantsci.2012.03.009 22608523

[B77] Saibi W. Drira M. Yacoubi I. Feki K. Brini F. (2015a). Empiric, structural and in silico findings give birth to plausible explanations for the multifunctionality of the wheat dehydrin (dhn-5). Acta Physiol. Plant. 37, 1–8. 10.1007/s11738-015-1798-7

[B78] Saibi W. Feki K. Mahmoud R. B. Brini F. (2015b). Durum wheat dehydrin (dhn-5) confers salinity tolerance to transgenic arabidopsis plants through the regulation of proline metabolism and ros scavenging system. Planta 242, 1187–1194. 10.1007/s00425-015-2351-z 26105651

[B79] Sivamani E. Wraith J. M. Al-Niemi T. Dyer W. E. Ho T. D. Qu R. (2000). Improved biomass productivity and water use efficiency under water deficit conditions in transgenic wheat constitutively expressing the barley hva1 gene. Plant Sci. 155, 1–9. 10.1016/S0168-9452(99)00247-2 10773334

[B80] Skinner J. S. Zitzewitz J. V. Szücs P. Marquezcedillo L. Filichkin T. Amundsen K.. (2005). Structural, functional, and phylogenetic characterization of a large cbf, gene family in barley. Plant Mol. Biol. 59, 533–551. 10.1007/s11103-005-2498-2 16244905

[B81] Svensson J. Ismail A. M. Palva E. T. Close T. (2002). Dehydrins, in Cell and Molecular Responses to Stress, Vol. 3, Sensing, Signaling and Cell Adaptation, eds Storey K. B. Storey J. M. (Amsterdam: Elsevier Press), 155–171.

[B82] Svensson J. T. Crosatti C. Campoli C. Bassi R. Stanca A. M. Close T. J.. (2006). Transcriptome analysis of cold acclimation in barley albina and xantha mutants. Plant Physiol. 141, 257–270. 10.1104/pp.105.072645 16603669 PMC1459312

[B83] Taulavuori E. Hellstrom E. K. Taulavuori K. Laine K. (2001). Comparison of two methods used to analyse lipid peroxidation from *Vaccinium myrtillus* (L.) during snow removal, reacclimation and cold acclimation. J. Exp. Bot. 52, 2375–2380. 10.1093/jexbot/52.365.2375 11709587

[B84] Tripepi M. Pöhlschroder M. Bitonti M. B. (2011). Diversity of dehydrins in oleae europaea plants exposed to stress. Open Plant Sci. J. 5, 9–13. 10.2174/1874294701105010009

[B85] Tunnacliffe A. Wise M. J. (2007). The continuing conundrum of the lea proteins. Sci. Nat. 94, 791–812. 10.1007/s00114-007-0254-y 17479232

[B86] Ukaji N. Kuwabara C. Takezawa D. Arakawa K. Fujikawa S. (2001). Cold acclimation-induced wap27 localized in endoplasmic reticulum in cortical parenchyma cells of mulberry tree was homologous to group 3 late-embryogenesis abundant proteins. Plant Physiol. 126, 1588–1597. 10.1104/pp.126.4.1588 11500557 PMC117158

[B87] Velten J. Oliver M. J. (2001). Tr288, a rehydrin with a dehydrin twist. Plant Mol. Biol. 45, 713–722. 10.1023/A:1010659120984 11430433

[B88] Wang X. Q. Yang P. F. Liu Z. Liu W. Z. Hu Y. Chen H.. (2009). Exploring the mechanism of physcomitrella patens desiccation tolerance through a proteomic strategy. Plant Physiol. 149, 1739–1750. 10.1104/pp.108.131714 19211702 PMC2663739

[B89] Waterer D. Benning N. T. Wu G. Luo X. Liu X. Gusta M. (2010). Evaluation of abiotic stress tolerance of genetically modified potatoes (*Solanum tuberosum*, cv. desiree). Mol. Breed. 25, 527–540. 10.1007/s11032-009-9351-2

[B90] Weigel D. Glazebrook J. (2002). Chapter 1: how to grow Arabidopsis, in Arabidopsis: a Laboratory Manual (Cold Spring Harbor; New York, NY: Cold Spring Harbor Laboratory Press), 1–17.

[B91] Welin B. V. Åke O. Nylander M. Palva E. T. (1994). Characterization and differential expression of dhn/lea/rab -like genes during cold acclimation and drought stress in *Arabidopsis thaliana*. Plant Mol. Biol. 26, 131–144. 7948863 10.1007/BF00039526

[B92] Wen Y. Wang X. Xiao S. Wang Y. (2012). Ectopic expression of vpaldh2b4, a novel aldehyde dehydrogenase, gene from chinese wild grapevine (*Vitis pseudoreticulata*), enhances resistance to mildew pathogens and salt stress in arabidopsis. Planta 236, 525–539. 10.1007/s00425-012-1624-z 22437646

[B93] Wise M. J. Tunnacliffe A. (2004). Popp the question: what do lea proteins do?. Trends Plant Sci. 9, 13–17. 10.1016/j.tplants.2003.10.012 14729214

[B94] Wolken J. J. Schwertz F. A. (1953). Chlorophyll monolayers in chloroplasts. J. Gen. Physiol. 37, 111−120 10.1085/jgp.37.1.111 13084895 PMC2147422

[B95] Xie Y. Xu D. Cui W. Shen W. (2012). Mutation ofarabidopsis hy1causes uv-c hypersensitivity by impairing carotenoid and flavonoid biosynthesis and the down- regulation of antioxidant defence. J. Exp. Bot. 63, 3869–3883. 10.1093/jxb/ers078 22419743 PMC3388838

[B96] Xu D. Duan X. Wang B. Hong B. Ho T. Wu R. (1996). Expression of a late embryogenesis abundant protein gene, hva1, from barley confers tolerance to water deficit and salt stress in transgenic rice. Plant Physiol. 110, 249–257. 10.1104/pp.110.1.249 12226181 PMC157716

[B97] Xu J. Tian Y. S. Peng R. H. Xiong A. S. Zhu B. Jin X. F.. (2010). Atcpk6, a functionally redundant and positive regulator involved in salt/drought stress tolerance in arabidopsis. Planta 231, 1251–1260. 10.1007/s00425-010-1122-0 20217124

[B98] Xue G. P. Way H. M. Richardson T. Drenth J. Joyce P. A. McIntyre C. L. (2011). Overexpression of TaNAC69 leads to enhanced transcript levels of stress up-regulated genes and dehydration tolerance in bread wheat. Mol. Plant 4, 697–712. 10.1093/mp/ssr013 21459832

[B99] Yamaguchi-Shinozaki K. Shinozaki K. (2006). Transcriptional regulatory networks in cellular responses and tolerance to dehydration and cold stresses. Plant Biol. 57, 781–803. 10.1146/annurev.arplant.57.032905.105444 16669782

[B100] Yoon J. Y. Hamayun M. Lee S.-K. Lee I.-J. (2009). Methyl jasmonate alleviated salinity stress in soybean. J. Crop Sci. Biotechnol. 12, 63–68. 10.1007/s12892-009-0060-5

[B101] Zhu J. K. Hasegawa P. M. Bressan R. A. (1997). Molecular aspects of osmotic stress in plants. Crit. Rev. Plant Sci. 16, 253–277. 10.1080/07352689709701950

