# Corrigendum: Physcomitrella Patens Dehydrins (PpDHNA and PpDHNC) Confer Salinity and Drought Tolerance to Transgenic Arabidopsis Plants

**DOI:** 10.3389/fpls.2018.00919

**Published:** 2018-07-03

**Authors:** Qilong Li, Xiaochen Zhang, Qiang Lv, Dong Zhu, Tianhang Qiu, Yu Xu, Fang Bao, Yikun He, Yong Hu

**Affiliations:** College of Life Sciences, Capital Normal University, Beijing, China

**Keywords:** dehydrins, Physcomitrella patens, salinity stress, drought tolerance, transgenic Arabidopsis plants

**Original text**

A DHN gene knockout *P. patens* mutant showed poorer recovery from salt and osmotic stress compared to the wild-type (WT), indicating that DHNs are indispensable for the alleviation of cell dehydration (Saavedra et al., 2006).

**Modified text**

A DHN gene knockout *P. patens* mutant showed poorer stress resistance and recovery from salt and osmotic pressure compared to the wild-type (WT), indicating that DHNs are indispensable for the alleviation of cell dehydration (Saavedra et al., 2006; Ruibal et al., 2012).

**Original text**

DHN genes are highly expressed under conditions of various types of stress, such as drought, cold, or high salinity (Yoon et al., 2009).

**Modified text**

DHN genes are highly expressed under conditions of various types of stress, such as drought, cold, or high salinity (Yoon et al., 2009; Agarwal et al., 2016).

In the original article, we neglected to cite two previous studies. The authors apologize for this oversight. This error does not change the scientific conclusions of the article in any way.

The original article has been updated.

## Conflict of interest statement

The authors declare that the research was conducted in the absence of any commercial or financial relationships that could be construed as a potential conflict of interest.

## References

[B1] Agarwal T. Upadhyaya G. Halder T. Mukherjee A. Majumder A. L. Ray S. (2016). Different dehydrins perform separate functions in physcomitrella patens. Planta 245, 1–18. 10.1007/s00425-016-2596-1 27638172

[B2] Ruibal C. Salamó I. P. Carballo V. Castro A. Bentancor M. Borsani O.. (2012). Differential contribution of individual dehydrin genes from physcomitrella patens to salt and osmotic stress tolerance. Plant Sci. Int. J. Exp. Plant Biol. 190, 89–102. 10.1016/j.plantsci.2012.03.009 22608523

[B3] Saavedra L. Svensson J. Carballo V. Izmendi D. Welin B. Vidal S. (2006). A dehydrin gene in physcomitrella patens is required for salt and osmotic stress tolerance. Plant J. 45, 237–249. 10.1111/j.1365-313X.2005.02603.x 16367967

[B4] Yoon J. Y. Hamayun M. Lee S.-K. Lee I.-J. (2009). Methyl jasmonate alleviated salinity stress in soybean. J. Crop Sci. Biotechnol. 12, 63–68. 10.1007/s12892-009-0060-5

