# Supplementary figures and images for: Physcomitrella Patens Dehydrins (PpDHNA and PpDHNC) Confer Salinity and Drought Tolerance to Transgenic Arabidopsis Plants
Source: Front Plant Sci. 2017 Jul 26;8:1316. doi: 10.3389/fpls.2017.01316 (PMC5526925; doi:10.3389/fpls.2017.01316)

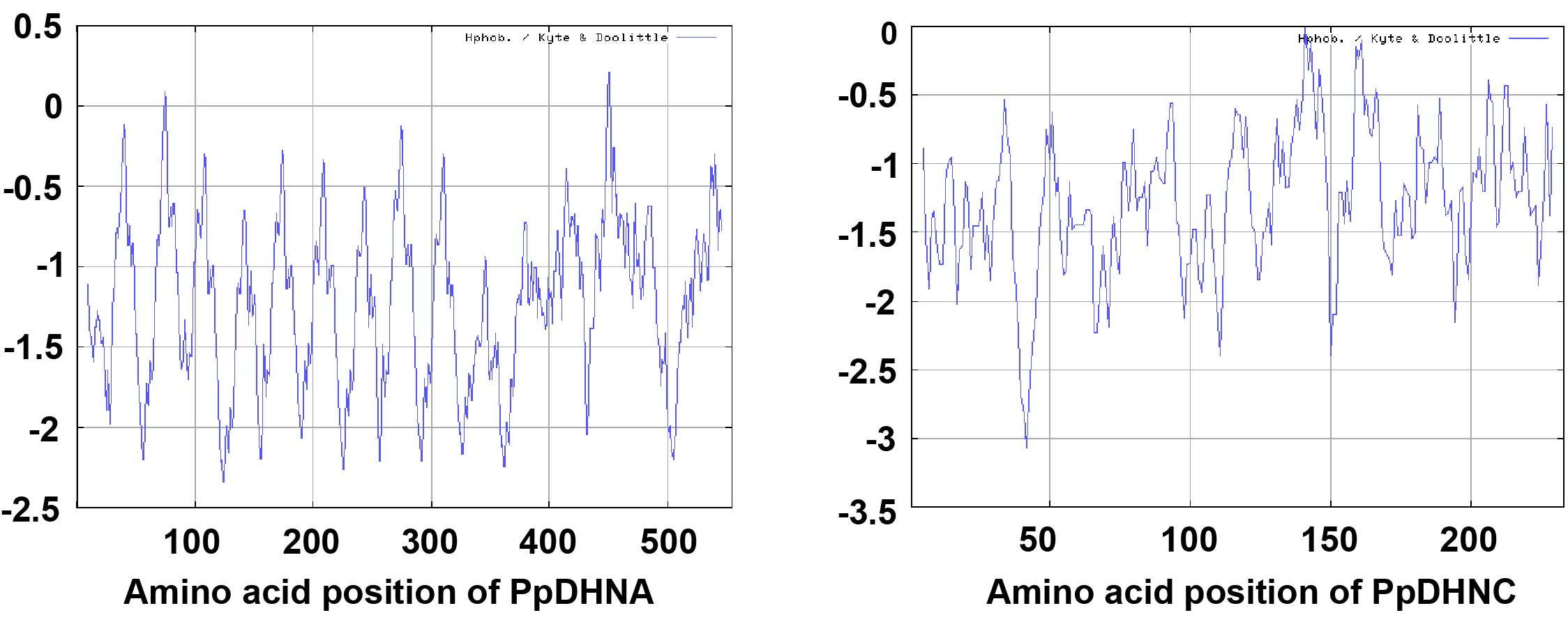

Supplement: Figure S1 — Kyte and Doolittle hydropathy analysis of PpDHNA and PpDHNC. Values below the zero lines are negative and hydrophilic, based on the average over a moving window of 19 amino acids. The numbers on the horizontal axis refer to the amino acid positions. [file Image1.JPEG]

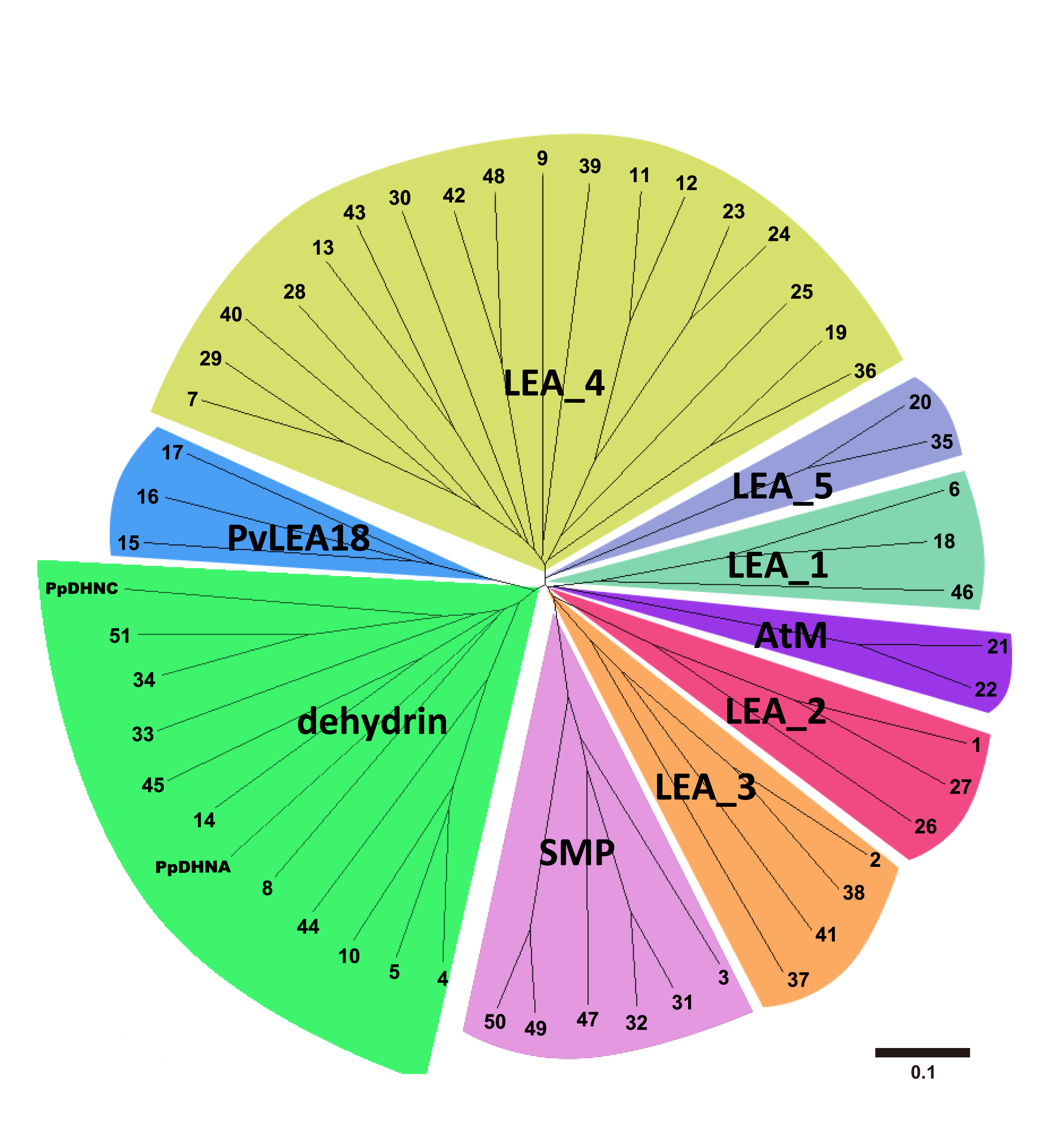

Supplement: Figure S2 — Unrooted dendrogram of all Arabidopsis LEA genes and PpDHNA and PpDHNC. Sequence alignments were performed unsing the ClustalW algorithm and an unrooted dendrogram was drawn subsequently. The different LEA groups are indicated by different colors. [file Image2.JPEG]

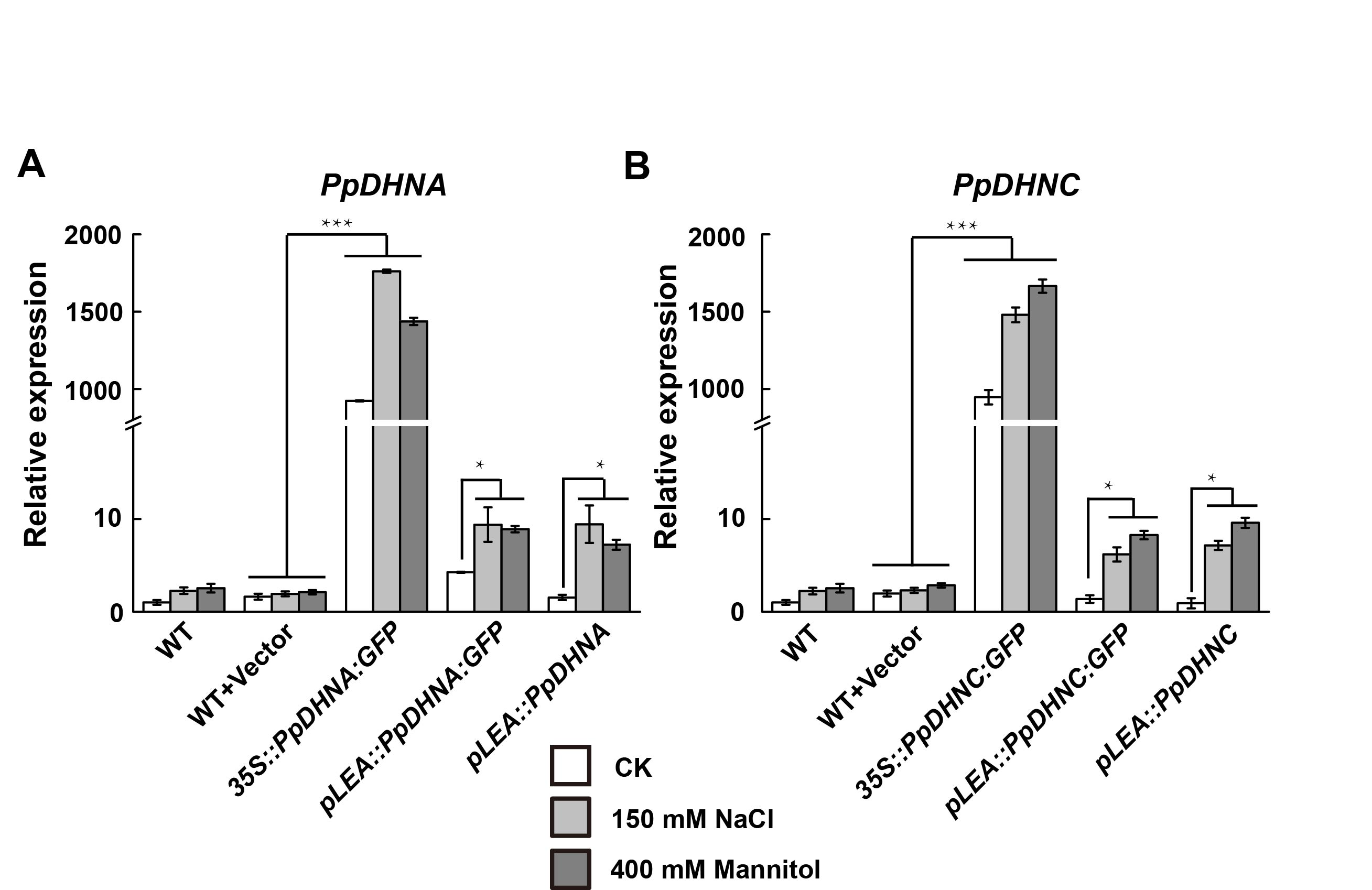

Supplement: Figure S3 — Salinity and osmotic stresses induced the transcriptional levels of PpDHNA and PpDHNC in transgenic plants. (A,B) Salinity-induced and osmotic-induced expression of PpDHNA and PpDHNC in the transgenic plants, analyzed using quantitative real-time PCR. Seedlings treated as in Figure 3A with 150 mM sodium chloride or 400 mM Mannitol for 24 h. Data are mean values ±SD, with three biological replicates for each sample. Data are statistically analyzed with one-way ANOVA (LSD and Tamhane). Asterisks indicate significant different (*P < 0.05; **P < 0.01; ***P < 0.001). [file Image3.JPEG]

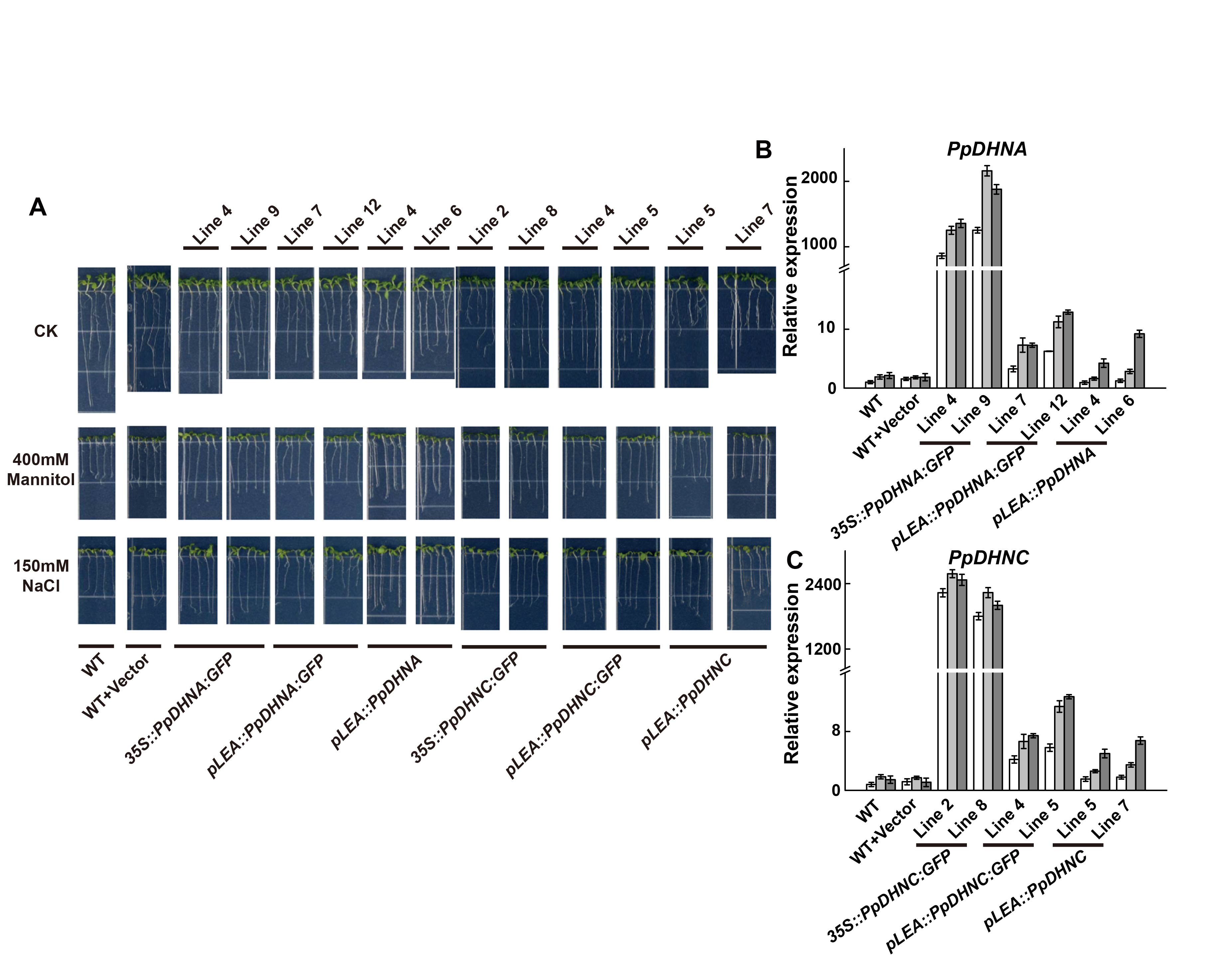

Supplement: Figure S4 — Effects of salinity and osmotic stresses on the others lines of PpDHNA and PpDHNC transgenic Arabidopsis seedlings. (A) Phenotypic comparison of wild type and PpDHN transgenic seedlings treated as indicated for 7 days. (B,C) Salinity-induced and osmotic-induced expression of PpDHNA and PpDHNC in the transgenic plants, analyzed using quantitative real-time PCR. Seedlings treated as in (A) with 150 mM sodium chloride or 400 mM Mannitol for 24 h. [file Image4.JPEG]

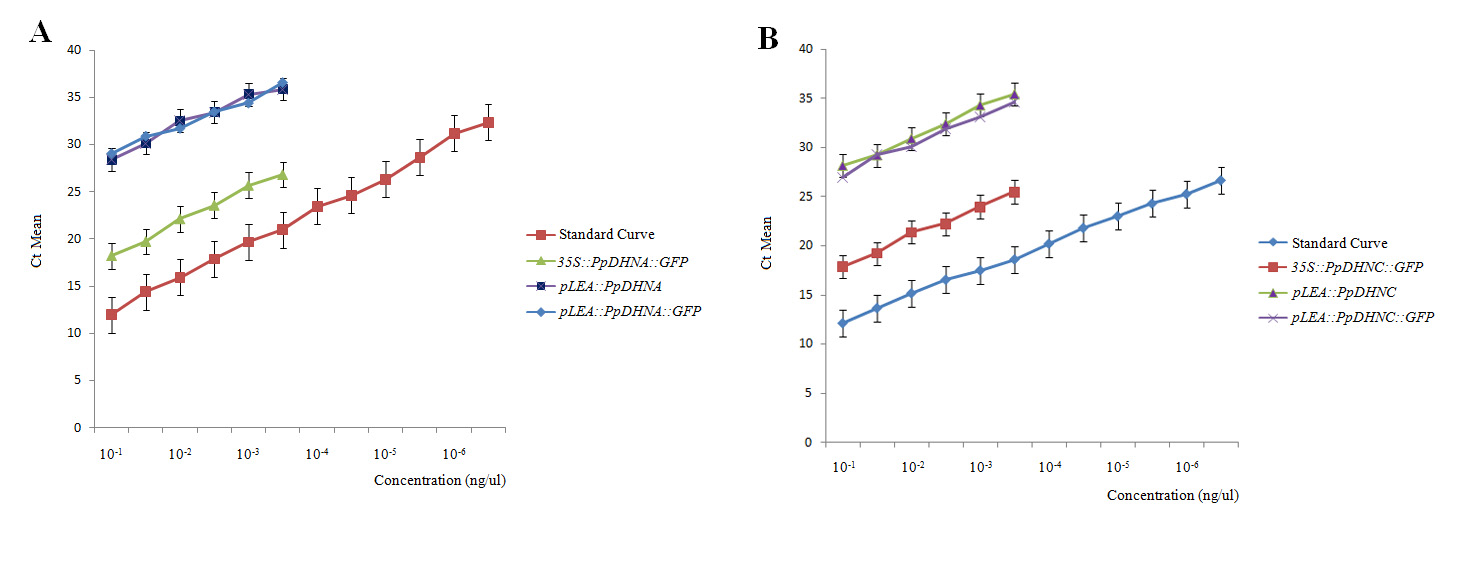

Supplement: Figure S5 — QRT-PCR analysis for copy number of genes. To plot standard curves, single copy vector was diluted to different concentrations. So did transgenic plants. By comparing the slope, each lines has a copy number similar to that of single copy vectors'. [file Image5.JPEG]

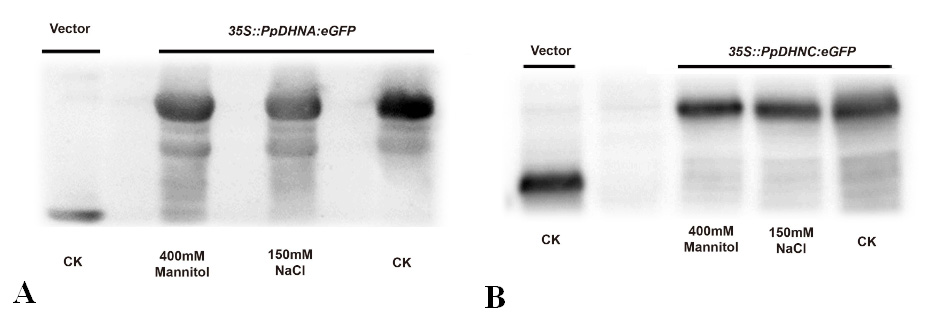

Supplement: Figure S6 — PpDHNA and PpDHNC protein expression under stresses. Vector and PpDHN transgenic seedlings treated as Figure 3 for 7 days. Salinity-induced and osmotic-induced expression of PpDHNA and PpDHNC proteins in the transgenic plants, analyzed using western blot. Because other lines suffer severe stress, they cannot grow normally. The accumulated protein could not be detected. Therefore, only the expression of 35S::PpDHNA::GFP and 35S::PpDHNC::GFP transgenic lines were shown. [file Image6.JPEG]

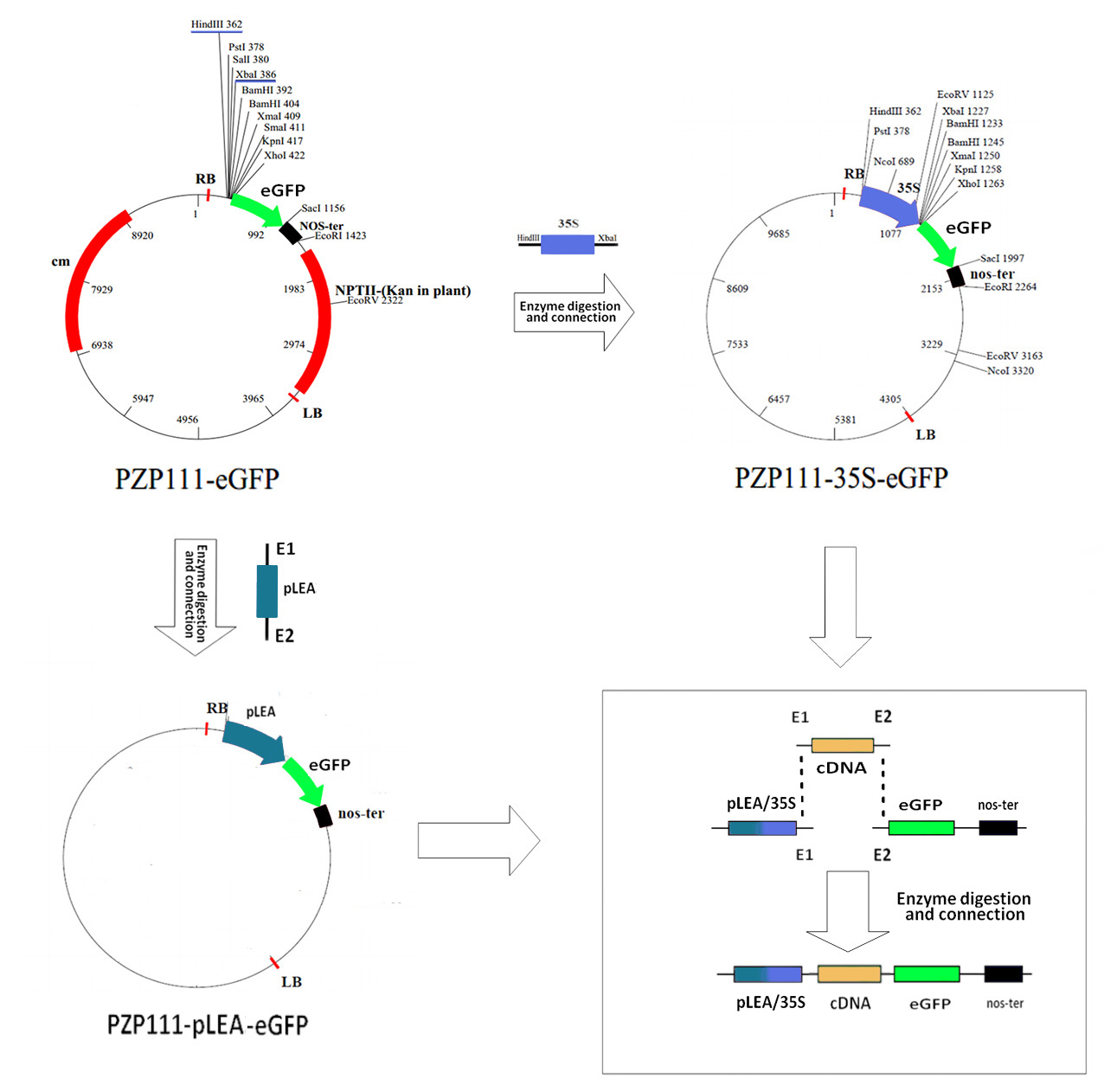

Supplement: Figure S7 — Gene constructs prepared in different vectors. [file Image7.JPEG]

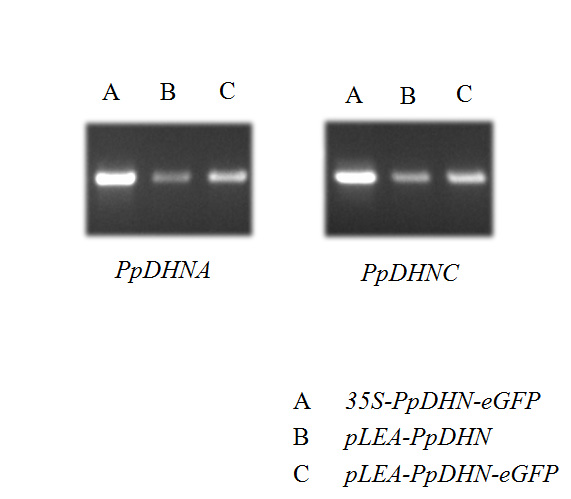

Supplement: Figure S8 — PCR analysis of the transgenic plants. [file Image8.JPEG]
