# Supplementary material for: Physcomitrella Patens Dehydrins (PpDHNA and PpDHNC) Confer Salinity and Drought Tolerance to Transgenic Arabidopsis Plants
Source: Front Plant Sci. 2017 Jul 26;8:1316. doi: 10.3389/fpls.2017.01316 (PMC5526925; doi:10.3389/fpls.2017.01316)
Supplement: Table S4 — Copy numbers of different genes in disfferent transgenic plants. [file Table4.DOCX]

| **line** | **Number of cDNA copies** |
| --- | --- |
| *35S-**PpDHNA-eGFP* | 6.87×10^6^±7.42×10^5^ |
| *35S-PpDHNC-eGFP* | 3.58×10^6^±1.27×10^6^ |
| *pLEA-PpDHNA* | 6.48×10^3^±1.45×10^3^ |
| *pLEA-PpDHNC* | 2.72×10^3^±6.36×10^2^ |
| *pLEA-PpDHNA-eGFP* | 7.89×10^3^±8.01×10^2^ |
| *pLEA-PpDHNC-eGFP* | 2.11×10^3^±2.32×10^2^ |

Absolute quantification of the mRNA expression level of duplicated *PpDHN* genes. Quantification was according to the *PpDHN* genes of the single copy vector in the quantitative real-time polymerase chain reaction (qRT-PCR) analysis. The values listed are indicated as cDNA copies/μg of reverse-transcribed total RNA, and the data are shown as the mean ± standard deviation.
